# Precipitation and local environment shape the geographic variation of seed size across natural populations of sand rice (*Agriophyllum squarrosum*)

**DOI:** 10.1093/jxb/erac231

**Published:** 2022-05-23

**Authors:** Pengshan Zhao, Xiaofeng Li, Ruilan Ran, Hong Sun, Jiecai Zhao, Guoxiong Chen

**Affiliations:** Key Laboratory of Stress Physiology and Ecology in Cold and Arid Regions, Gansu Province, Northwest Institute of Eco-Environment and Resources, Chinese Academy of Sciences, Lanzhou 730000, P.R. China; Shapotou Desert Research and Experiment Station, Northwest Institute of Eco-Environment and Resources, Chinese Academy of Sciences, Lanzhou 730000, P.R. China; Gaolan Station of Agricultural and Ecological Experiment, Northwest Institute of Eco-Environment and Resources, Chinese Academy of Sciences, Lanzhou 730000, P.R. China; Key Laboratory of Stress Physiology and Ecology in Cold and Arid Regions, Gansu Province, Northwest Institute of Eco-Environment and Resources, Chinese Academy of Sciences, Lanzhou 730000, P.R. China; University of Chinese Academy of Sciences, Beijing 100049, P.R. China; Key Laboratory of Stress Physiology and Ecology in Cold and Arid Regions, Gansu Province, Northwest Institute of Eco-Environment and Resources, Chinese Academy of Sciences, Lanzhou 730000, P.R. China; University of Chinese Academy of Sciences, Beijing 100049, P.R. China; Key Laboratory of Stress Physiology and Ecology in Cold and Arid Regions, Gansu Province, Northwest Institute of Eco-Environment and Resources, Chinese Academy of Sciences, Lanzhou 730000, P.R. China; University of Chinese Academy of Sciences, Beijing 100049, P.R. China; Key Laboratory of Stress Physiology and Ecology in Cold and Arid Regions, Gansu Province, Northwest Institute of Eco-Environment and Resources, Chinese Academy of Sciences, Lanzhou 730000, P.R. China; Shapotou Desert Research and Experiment Station, Northwest Institute of Eco-Environment and Resources, Chinese Academy of Sciences, Lanzhou 730000, P.R. China; Key Laboratory of Stress Physiology and Ecology in Cold and Arid Regions, Gansu Province, Northwest Institute of Eco-Environment and Resources, Chinese Academy of Sciences, Lanzhou 730000, P.R. China; Shapotou Desert Research and Experiment Station, Northwest Institute of Eco-Environment and Resources, Chinese Academy of Sciences, Lanzhou 730000, P.R. China; University of Massachusetts Amherst, USA

**Keywords:** *Agriophyllum squarrosum*, desert ecosystem, geographic pattern, intraspecific trait variation, precipitation gradient, phenotypic plasticity, sand rice, seed size

## Abstract

Sand rice (*Agriophyllum squarrosum*) is widely distributed on dunes in the Asian interior arid zone, and its large intraspecific trait variation makes it a very good model for investigating the ecological processes underlying its adaptation to the desert environment. In this study, seed size variation across 68 natural populations was used to establish geographic patterns and to quantify the effects of the climate, soil, and collection-year weather variables. The length of the seed major axis and thousand seed weight (TSW) both showed significant longitudinal patterns. Long-term climate variables accounted for most of the explained variances for seed major axis (57.20%) and TSW (91.54%). Specifically, annual precipitation and minimum monthly precipitation had the most significantly positive and negative effects, indicating that longitudinal clines are driven by a precipitation gradient across the species’ distribution range. A substantial unique effect of soil variables (27.27%) was found for seed major axis variation, but only 3.64% of TSW variation was explained by soil variables. Two extreme groups were selected to evaluate the genetic and plastic effects on seed size in a common garden experiment. Large-seeded individuals were more competitive in semi-arid regions, and had stronger adaptive plasticity as well as better performance in early seedling establishment, and hence they have potential for use in future domestication projects.

## Teaser

Precipitation underlies the longitudinal cline of seed size across the distribution range of sand rice, and large-seeded individuals have strong adaptive plasticity that has potential for use in future domestication.

## Introduction

Seed size (mass) is a key functional trait for many aspects of plant evolutional fitness and it varies dramatically among species, spanning a range of eleven orders of magnitude (Harper *et al.*, 1970; Westoby *et al.*, 1992; Moles *et al.*, 2005b). This enormous diversity in seed size has interested ecologists and evolutionary biologists for decades and a wide range of ­theoretical and empirical research has been conducted. This has led to the emergence of a complex picture in which the evolution of seed size variation is determined by multiple environmental and ecological factors, genome size, seed dispersal syndromes, and growth form, and is coordinated by intricate molecular networks with a wide variety of genetic components (Salisbury, 1974; Leishman *et al.*, 1995; Moles *et al*., 2005a, 2005b; Beaulieu *et al.*, 2007; Linkies *et al.*, 2010; Leslie *et al.*, 2017; Li *et al.*, 2019).

Latitude usually co-varies with macroclimatic variables, especially temperature, and thus latitudinal gradients provide a promising system for unravelling the clinal patterns of geographic variation in seed size (De Frenne *et al.*, 2013). Indeed, large-scale interspecific and community studies have revealed a negative relationship between seed size variation and latitudinal gradients, with seed size increasing significantly towards the equator (Moles and Westoby, 2003; Moles *et al.*, 2007; Gallagher and Leishman, 2012; Swenson *et al.*, 2012). However, intraspecific seed size variation across latitudinal gradients exhibits more complex patterns. Moles and Westoby (2003) reported that seed size within species decreases significantly towards the poles, but by 3.6-fold less than the decrease observed between species. A meta-analysis by De Frenne *et al.* (2013) found that latitude is the most crucial explanatory variable in the intraspecific trend of seed size, and that seed size substantially declines with latitude within natural populations of species. A similar latitude pattern has also been found in *Glycine* species in Australia with seed size being larger at lower latitudes, whilst temperature and solar radiation are the major predictors of seed size variation within the species’ range (Murray *et al.*, 2004). However, this negative pattern is not always observed when the latitudinal range is smaller, and some studies on forest herbs have shown either no intraspecific patterns between seed size and latitude (De Frenne *et al*., 2010, 2011) or a positive pattern (Graae *et al.*, 2009). Thus, current evidence suggests that geographic patterns in intraspecific seed size are inconsistent among individual species, and cannot be simply extrapolated from interspecific patterns (Moles and Westoby, 2003; Soper Gorden *et al.*, 2016; Moles, 2018).

Correlations between latitude and temperature are obscured by the fact that other climate and local environmental factors also co-vary with latitude, such as soil fertility soil physical and chemical properties, and water availability (De Frenne *et al.*, 2013). For example, weather variables in the year of seed collection are the best predictors of size in annual species (Soper Gorden *et al.*, 2016), and accumulated growing degree-hours are positively related to seed size in understorey forest herbs (De Frenne *et al.*, 2010). Positive relationships are seen between seed size and soil pH and solar radiation, whereas soil moisture is negatively correlated with seed size (Murray *et al.*, 2004; Tautenhahn *et al.*, 2008; Simpson *et al.*, 2016). Seed size is also larger in soils with higher clay contents (Dubuis *et al.*, 2013). Moreover, precipitation co-varies with temperature in complex patterns across spatial scales, and inconsistent trends ­between precipitation and seed mass have been reported (Baker, 1972; Murray *et al.*, 2004; Gallagher and Leishman, 2012; Moles *et al.*, 2014). Therefore, using latitude as a main proxy alone is insufficient to explain the temperature effects along latitudinal gradients (De Frenne *et al.*, 2013).

Aridification and desertification of the Asian interior are the results of uplift of the Tibetan Plateau, global cooling, and the westward retreat of the Paratethys Sea during the Cenozoic era (Fang *et al.*, 2002; Miao *et al.*, 2012; Bosboom *et al.*, 2014). The environment is highly vulnerable to climate change and human influences (Lu *et al.*, 2019), but despite this desert plants from Asian interior have rarely been the subject of global or regional research. Sand rice (*Agriophyllum squarrosum*, Amaranthaceae) is widely distributed and adapted to mobile sand dunes in Central Asia, and has been promoted as having potential for development as a crop since its nutritional value is comparable with that of quinoa (*Chenopodium quinoa*) (Chen *et al.*, 2014; Zhao *et al*., 2021). As a pioneer species, its establishment of populations paves the way for the invasion of other species, thereby initiating the vegetative restoration of active sand dunes in arid regions (Nemoto and Lu, 1992; Zhang *et al.*, 2003). Its broad geographic range and ecological significance make sand rice a good model for studying intraspecific variation of seed size in response to climate change and the desert environment.

Previous common garden experiments have demonstrated that sand rice populations have high variances in plant height, stem diameter, basal branch length, above-ground biomass, and seed weight and diameter (Yin *et al*., 2016a, 2016b; Zhang *et al.*, 2018). This intraspecific variation enables acclimation and/or local adaptation to diverse niches with differing climatic and local environmental variables, and hence the broad ecological distribution of the species. A redundancy analysis has shown positive and/or negative latitudinal patterns of most traits among 26 populations that were derived from the eastern sand fields and middle deserts in northern China (Yin *et al.*, 2016b). Of these traits, seed diameter increased at higher latitudes and was positively correlated with the seasonality and annual range of temperature. Interestingly, seed diameter was also reported to vary along latitude and longitude gradients, and positively correlated with the maximum wind speed in winter for 16 out of 26 populations that were studied (Yin *et al.*, 2016a). In addition, simulation experiments in the Mu Us sand field of China have shown that the amount and frequency of precipitation are positively correlated with the seed mass variation of sand rice (Gao *et al.*, 2014). Because previous studies have focused on restricted geographic ranges and a limited number of populations, the patterns of seed size variation in sand rice across greater spatial scales and with wider variations in abiotic stress remain unknown. More studies with careful experimental design and appropriate statistical tools, such as multiple regression and variance partitioning (De Frenne *et al.*, 2013), are required to disentangle the effects of geographic, climatic, and local environmental variables on seed size variation across natural populations. In the current study, the geographic pattern of seed size variation was examined across 68 natural populations from Kazakhstan and China, covering most of the ecological range of sand rice. The effects of long-term climate and local environment variables were determined using multiple regression and variance partitioning. In addition, a common garden experiment was conducted to examine the role of phenotypic plasticity in seed size variation.

## Materials and methods

### Sampling of natural sand rice populations

A total of 68 natural populations of sand rice (*Agriophyllum squarrosum*) were sampled between 2013 and 2018 in China and Kazakhstan a region between approximately 30–50°N and 75–125°E (Supplementary Table S1). For each population, seeds were harvested from independent mature individuals at least 30 m apart between September and the following February. The seeds were stored in refrigerator at -20 °C for the following experiment. A total of 871 individuals across the populations were used to measure the length of the major axis and the thousand seed weight (TSW) (Supplementary Fig. S1; Supplementary Table S1). For each individual, 5–20 seeds were used for measuring the major axis with ImageJ. Either 100 or 200 seeds were weighed and used to calculate the TSW for 16 populations. For 48 populations, 50 seeds were measured with 2–4 replicates. Since there were few seeds in the four Kazakhstan populations, the TSW values were predicted as described below. The regression models between TSW and major axis length at the population and individual levels were constructed using the *basicTrendline* R package (https://CRAN.R-project.org/package=basicTrendline).

### Geographic, climatic, and soil data, and weather data for the seed collection year

The latitude and longitude for each population was recorded by GPS. The elevation data were extracted based on geographic information using the *raster* R package (https://CRAN.R-project.org/package=raster). Nineteen climate variables for each population were downloaded from WorldClim2 with 30-s spatial resolution (https://worldclim.org). Soil variables were extracted from SoilGrids (https://soilgrids.org) and the Harmonized World Soil Database v1.2 (HWSD, http://webarchive.iiasa.ac.at/Research/LUC/External-World-soil-database/HTML/). Aridity index (AI) and potential evapotranspiration (PET) were downloaded from the Global Aridity Index and Potential Evapo-Transpiration (ET0) Climate Database v2 (https://cgiarcsi.community). Since the values from Global-Aridity_ET0 represent the moisture availability, the aridity level for each site was calculated as 10 000–AI in Supplementary Table S1. Triangle plots of soil textures were generated using the *plotrix* R package (Lemon, 2006). Moisture index (MI) was calculated as minimum monthly precipitation (PPTmin)/potential evapotranspiration (PET). The nearest weather station for each population was used to obtain the seed collection-year weather information. The temperature and precipitation data from the previous October to the October of the collection year were selected to calculate the PPTmin, maximum monthly precipitation (PPTmax), total monthly precipitation (PPTtotal), coefficient of variation of monthly precipitation (PPTcv), and number of days with daily temperature above 5 °C (TMP5nb).

### Estimation of missing values

The four populations located in Kazakhstan only contained one individual each, and TSW values and collection-year weather data were not available. In addition, the YJ_YuanZ population from China did not have very good seed quality, and although a mean TSW value of 1.28 g was obtained, it was also set as ‘not available’. To generate predictions for these missing data, the whole dataset of the 68 populations including individual numbers, major axis length values, TSW values, 19 climate variables, PET, AI, 17 soil variables, and five collection-year weather indices was used as input data for the *knnImputation* function in the *DMwR* package (Torgo, 2010), with default setting (*k* = 10). For example, the predicted value for TSW of the YJ_YuanZ population was 0.82 g, which was very similar to that of two nearby populations (YJ_XY, 0.88 g; YJ_YZ, 0.72 g; Supplementary Table S1), suggesting that the simulation method worked well. Consequently, the new, complete dataset with predicted values was used in following analyses.

### Species distribution model

The *dismo* R package (https://rspatial.org/raster/sdm/index.html) was employed to predict the distribution of sand rice. In brief, sampling bias was removed by setting the resolution of the cells to 0.5 degrees. The collinearity in 19 climate variables (listed in Supplementary Table S1) was detected using the *usdm* R package (Naimi *et al.*, 2014) and a set of predictor variables with low variance inflation factors were automatically obtained by setting the threshold value at 0.85. A total of 51 occurrence sites and the following eight climate variables were used to predict the suitable habitats of sand rice: mean diurnal range (Bio02), temperature seasonality (Bio04), mean temperature of wettest quarter (Bio08), mean temperature of driest quarter (Bio09), precipitation of driest month (Bio14), precipitation seasonality (Bio15), precipitation of warmest quarter (Bio18), and precipitation of coldest quarter (Bio19). Seven implemented algorithms were fitted with the environmental data, namely Bioclim, Domain, Mahalanobis distance (Mahal), Generalized linear models, Maxent, Random Forest, and Support vector machines, and the individual model predictions were stacked together. After comparing with the true occurrence records, only the predictions by Mahal and Maxent were retained. The two predictions were combined by applying the model average scores.

### Clustering analyses, correlations, and principal component analysis

To understand the relationship between seed size variation and the geographic distribution of the sand rice populations at a coarse level, the eight climate variables listed above and elevation data were employed as proxies to construct a hierarchical cluster heatmap using the *pheatmap* R package with default settings (https://CRAN.R-project.org/package=pheatmap). The columns were annotated by group names (A–G) and the deserts where each population was sampled (Supplementary Table S1).

Collinearity among predictor variables will affect the stability of the final results in regression models. In our species distribution modeling, the final variables were selected through a stepwise procedure. To find the biologically meaningful variables, pairwise correlations among geographic, climate, soil, and collection-year weather variables were performed using the *ggcorrplot* R package (https://CRAN.R-project.org/package=ggcorrplot). Individual correlations and *P*-values were also calculated for each of the pairs of variables. In addition, principal component analysis (PCA) was conducted for each of the three kinds of variables using the *FactoMineR* (Lê *et al.*, 2008) and *factoextra* R packages (https://CRAN.R-project.org/package=factoextra). The important variables with low collineartiy were selected by setting the threshold of correlation values at 0.7 and according to loading values on the PCA dimensions.

### Regression modeling

Linear models were constructed to investigate the effects of geographic variables on length of the major axis and TSW among the sand rice natural populations. Latitude and longitude data were included as ­predictor variables, whereas elevation data were excluded because of the high correlation with latitude. Linear mixed-effects models were also constructed to inspect the geographic effects using the *lme4* R package (Bates *et al.*, 2015). The group and collection-year were treated as random effects and statistical tests were performed using the *lmerTest* R package (Kuznetsova *et al.*, 2017). The best models were selected based on the corrected Akaike’s information criterion (AICc) and ANOVA results, and ‘1|collection-year/group’ and ‘1|group’ were finally used as random effects for the major axis length and TSW, respectively (Supplementary Fig. S2). Only longitude was the significant predictor for the major axis (*P*=0.013). The marginal *R*^2^ values for both models were 0.22 and 0.10, respectively. When longitude was included as the single predictor, similar results were obtained for both models (marginal *R*^2^ for major axis length was 0.21, *P*=0.01; marginal *R*^2^ for TSW was 0.11, *P*=0.15). Statistical tests using the ‘*ranova*’ function in the *lmerTest* R package showed that the random effects were significant in all models, whereas the estimated residual variance for each random factor was very close to zero. Therefore, random factors were ignored and only linear regression results are shown in the text. Similarly, the same linear model procedures were performed at the level of the individual (Supplementary Figs S3, S4). The *ggeffects* and *ggplot2* R packages (Wickham, 2016; Lüdecke, 2018) were respectively used to calculate and plot values for the main effect and marginal effect of longitude in the different models.

Multiple regression models were constructed according to Gross *et al.* (2017) to dissect the effects of the climate, soil, and collection-year weather variables on major axis length and TSW. Before modeling, all predictor variables were *z*-score standardized. The selection of best model was performed using the ‘*dredge*’ function of the *MuMIn* R package (https://CRAN.R-project.org/package=MuMIn), and the predictors were selected based on AICc (ΔAICc<2). Where multiple models were selected, they were averaged according to AICc weighting. In addition, the *step()* function and the *leaps* R package (https://CRAN.R-project.org/package=leaps) were used to validate the best models of major axis length and TSW. The relative importance of each variable on major axis or TSW was evaluated by calculating the parameter estimate for each predictor in each model. The contribution fractions of the climate, soil, and collection-year weather variables to the explained variations of major axis length and TSW were calculated against the total parameter estimates in corresponding models. This method is similar to variance partitioning since all variables were *z*-score transformed before modeling (Gross *et al.*, 2017).

### Seed size plasticity assay

After sorting the sand rice individuals according to the length of the seed major axis, those with the largest and smallest values were used for comparison. Each group contained 96 individuals, and 20 seeds from each individual were directly sown into pots filled with a 1:1 mixture of loess and nutrient soil (Pindstrup, Denmark) at Lanzhou on 7 May, 2019. The seeds were covered with 1 cm sand. From 6 d after sowing, seedling emergence and survival were recorded daily for 1 week, then every 5 d for the next 30 d, and finally at two further 20-d intervals (until 29 July). Plant height was measured when the plants were mature and the seeds were harvest and used to measure the major and minor axis lengths. A linear model was constructed to assess the interaction effect between environment (Natural versus Common garden) and seed size group (Large versus Small) on seed major axis length. The fitted model had an adjusted *R*^2^ value of 0.88 and an *F*-statistic *P*-value < 2.2 × 10^–16^. The trendline was generated using the *ggplot2* R package (Wickham, 2016).

### Statistical analysis

All data were analysed with R version 3.6 (www.r-project.org). For statistical analysis, the data were first tested for normality using *shapiro.test* and for homogeneity of variance using *bartlett.test*, and then were subjected either to *anova* or *kruskal.test* for multiple sets of data, or *wilcox.test* or *t.test* for pairwise comparisons. Histograms and boxplots were generated using the basic plot functions in R.

## Results

### Geographic range and seed size variation

**Fig. 1.**
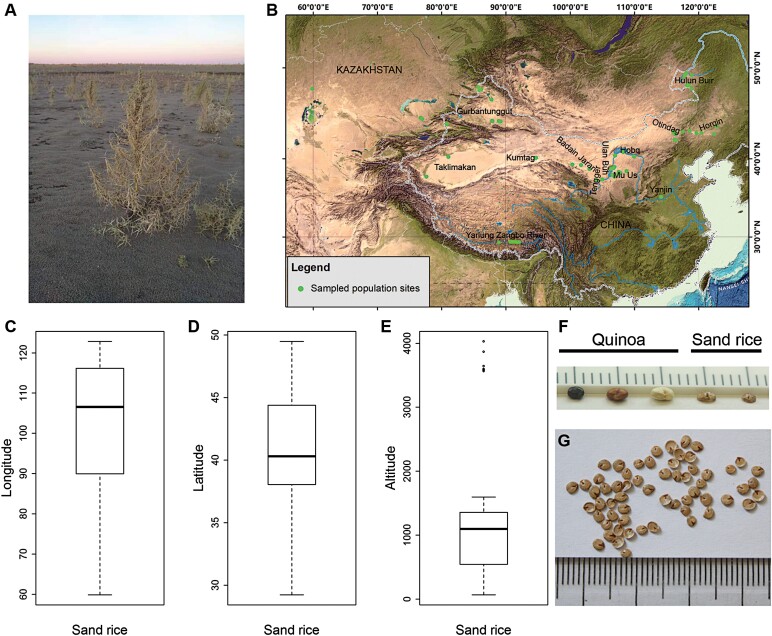
Geographic distribution of sand rice. (A) A mature sand rice plant in Xin Barag Left Banner, Hulun Buir. (B) Geographic distribution of sand rice in China and Kazakhstan. The background imagery is reproduced from GEBCO_2014 Grid, version20150318 (http://www.gebco.net). Boxplots of (C) longitude, (D) latitude, and (E) altitude of the 68 sand rice populations studied. (F) Comparison of seed size between quinoa and sand rice, and (G) seed phenotype of sand rice.

Sand rice (Fig. 1A) is mostly found in sand fields and deserts, including the Hulun Buir, Horqin, Otindag, and Mu Us sand fields, the Hobq, Ulan Buh, Tengger, Badain Jaran, Kumtag, Gurbantunggut, and Taklimakan deserts, and in some sand dunes by the Yarlung Zangbo and Yellow Rivers in China as well as deserts in Kazakhstan (Fig. 1B). Its geographic distribution is over a latitude range from 29.24–49.48°N and a longitude range from 59.88–122.84°E, and from 69–4029 m altitude (Fig. 1C–E). Sand rice seeds are mostly oval in shape and are smaller than those of quinoa (Fig. 1F, G). The length of the major axis was measured to represent the seed size in this study (Supplementary Fig. S1).

**Fig. 2.**
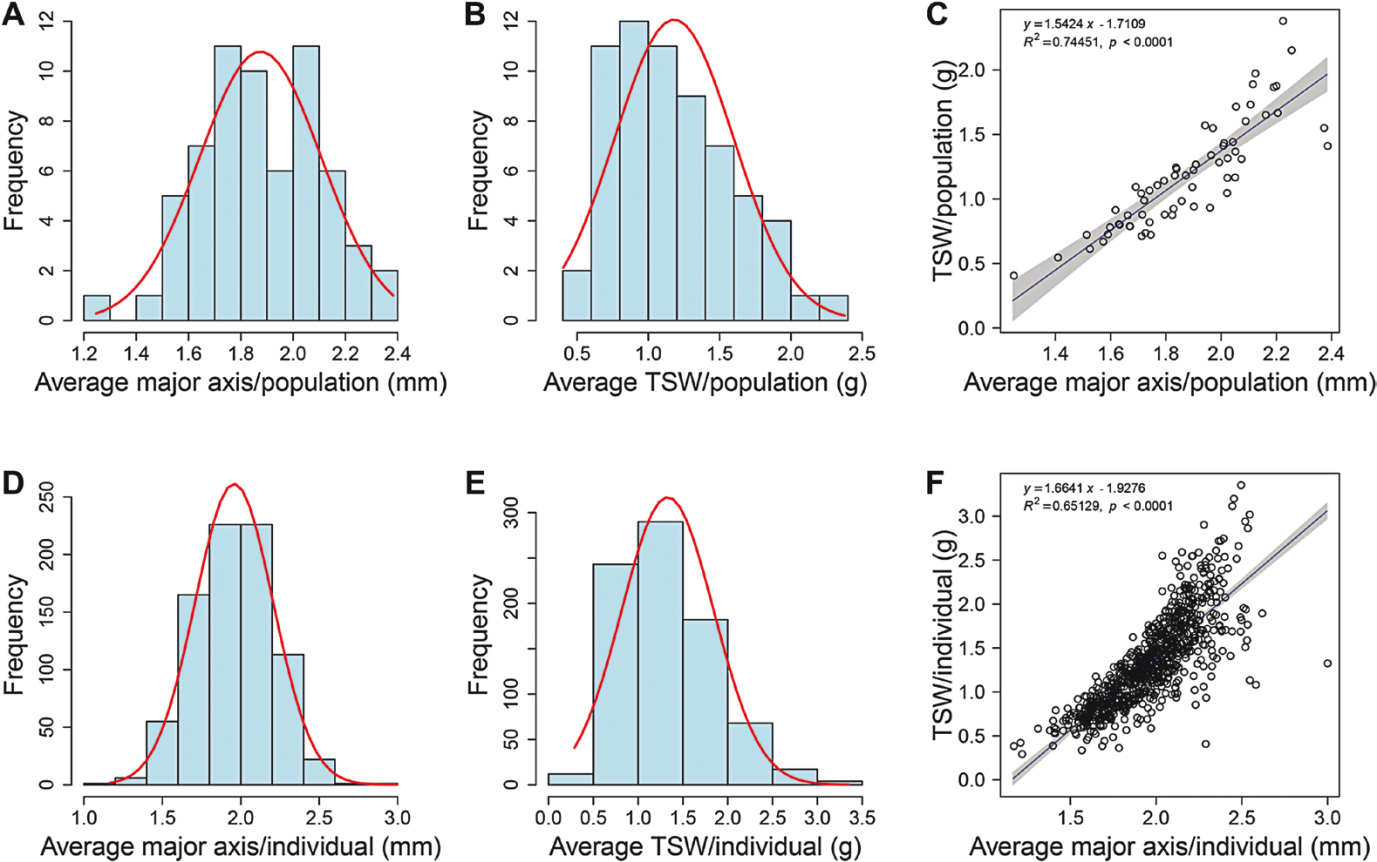
Frequency distributions of major axis length and thousand seed weight (TSW) of sand rice at the population and individual levels. (A) Mean major axis length and (B) TSW for 63 natural populations. (C) Regression of major axis length and TSW at the population level. (D) Mean major axis length and (E) TSW at the level of the individual. A total of 816 individuals were sampled across the 63 populations. (F) Regression of major axis length and TSW at the individual level. The lines in (A, B) and (D, E) are the fitted normal distribution curves. The regressions in (C, F) were generated using the *basicTrendline* R package.

A total of 68 natural populations were sampled across the sand rice distribution range, with the number of individuals in each population ranging from one to 32. A total of 871 individuals across all the populations were used to measure the seed major axis length. The average (mean) major axis length of populations was 1.87 mm, ranging from 1.25 to 2.38 mm, while the average value of individuals was 1.96 mm, ranging from 1.17 to 2.99 mm (Supplementary Fig. S5; Supplementary Table S1). A total of 818 individuals from 64 populations were used to determine thousand seed weights (TSW), which resulted in mean values for populations and individuals of 1.18 g (0.40–2.38 g) and 1.33 g (0.29–3.35 g), respectively. These results indicated that large intraspecific variance in seed size existed among the natural populations. We used the data from 816 individuals in 63 populations to conduct regression analyses, with the YJ_YuanZ population near the Yellow River comprising only one individual being excluded because of poor seed quality. Frequency distributions showed that the data were normally distributed (Fig. 2). A significant correlation was found between the average population major axis length and TSW (*R*^2^=0.74, *P*<0.0001; Fig. 2C), and a similar pattern was also observed at the level of the individual, although the relationship was not as strong (*R*^2^=0.65, *P*<0.0001; Fig. 2F).

### Seed size variation with longitude

After accounting for sampling bias, a total of 51 representative populations were selected and corresponding climate data were downloaded from the WorldClim database. The collinearity of 19 variables within each sample site was assessed by calculating variance inflation factor values, and eight were selected for use in species distribution modeling using *dismo* R package (see Supplementary Table S1). Out of seven models that we implemented, the Mahal and Maxent models and their combined model average achieved the best results, with all the known locations of sand rice occurrences being covered in the predicted distribution ranges (Supplementary Fig. S6). Hence, these eight variables were used for preliminary evaluation of the geographic and/or climatic clines of the sand rice trait variations.

**Fig. 3.**
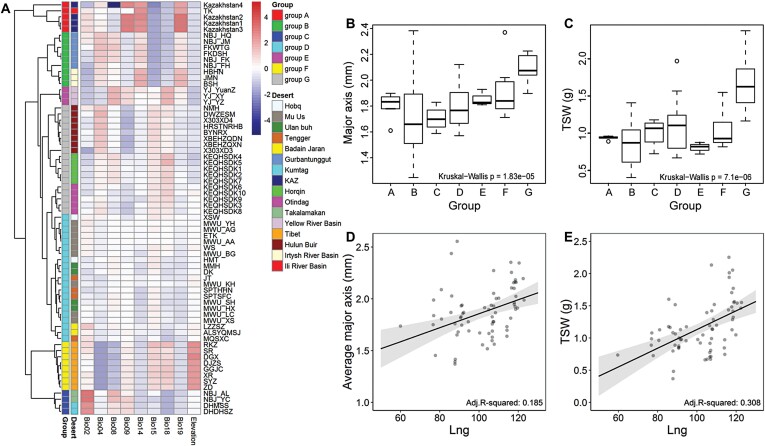
Hierarchical clustering of 68 natural populations of sand rice and the longitudinal pattern of seed size variation. (A) Heatmap of the populations based on eight climatic variables and elevation data. The populations are categorized into seven groups (A–G). The regional locations are also indicated. The populations are listed in Supplementary Table S1. (B, C) Boxplots showing variations among the the groups for (B) major axis length and (C) thousand seed weight (TSW). (D, E) Linear regression results of major axis length and TSW at the population level. Longitude and latitude were included as predictors and the adjusted predictions of longitude on (D) major axis length and (E) TSW are shown as determined using the *ggeffects* R package.

Hierarchical cluster analysis was performed with the eight climate variables and the elevation data of each of the 68 populations, and this resulted in classification into seven groups (A–G), that were well matched with the geographic origins (Fig. 3). For instance, group A included the populations from the Kazakhstan deserts and the Tukai (TK) desert in the Ili River Basin of west China, and group G included populations from the Otindag, Horqin, and Hulun Buir sand fields in northeast China (Fig. 3A). Re-analysis of the values of major axis length and TSW for each group indicated that seed sizes in the populations in the east (e.g. group G) were significantly higher than those in the west (e.g. group A; *P*<0.001), and major axis length exhibited a generally increasing trend from the west groups to the east groups (Fig. 3B, C). Linear regressions were used to test the effects of latitude and longitude, and the results shown that longitude was the significant geographic predictor for major axis length and TSW (*P*<0.001) in bivariate and multivariate linear models (Fig. 3D, E; Supplementary Fig. S7), whereas latitude was not (*P*=0.41 for major axis length; *P*=0.08 for TSW). The same regression processes were also conducted at the level of the individual and the results resembled those of the population level analyses (Supplementary Figs S3, S4). These results suggested that major axis length and TSW exhibit significant longitudinal (west-to-east) patterns in the natural range of sand rice, albeit only explaining a minor part of the variance (adjusted *R*^2^≤0.31).

### Quantifying the effects of climate and local environment on seed size variation

**Fig. 4.**
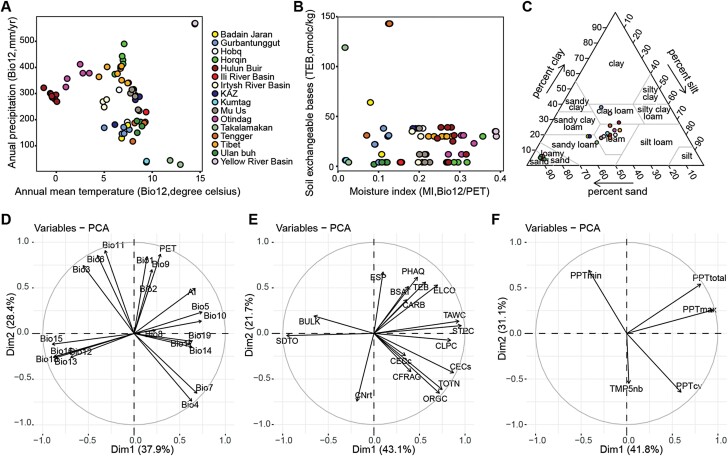
Influence of climate, soil, and collection-year weather variables on seed size variation in the natural sand rice populations. (A) Relationship between annual mean temperature and annual precipitation for the 68 populations. (B) Relationship between soil fertility (measured as exchangeable bases) and moisture index. (C) Triangle plot of soil texture at the 68 study sites. (D–F) Principal component analysis (PCA) of (D) long-term climatic variables, (E) soil variables, and (F) collection-year weather variables. Dim1 and Dim2 present the first and second principal components, respectively. The variables are described in Supplementary Table S1.

Among the populations, the annual mean temperature ranged from –1.31 to 14.48 °C, with most below 10 °C, while the annual precipitation ranged from 28 mm to 569 mm, with 57% of populations located in arid and extreme arid regions (<300 mm; Fig. 4A). Moisture indices averaged 0.02–0.39. Values for soil total exchangeable bases (TEB), which is an important index of soil fertility, were below 65 in most of the populations, and only four had values over 100 (Fig. 4B). In terms of soil texture, sand rice was found in soils with >34% sand and <41% silt and <38% clay (Fig. 4C), indicating that it can grow in sand, loamy sand, sandy loam, and loam, as well as clay loam soils.

To select the most important climate, soil, and collection-year weather variables, PCAs were conducted. Two significant PCs were identified, which explained 37.9% and 28.4% of the climatic variation among the 68 populations (Fig. 4D). All the precipitation variables either positively or negatively correlated with climate PC1 (*P*<0.01), with precipitation seasonality (Bio15) as the most strongly loaded variable (*P*=6.97 × 10^–25^). The temperature variables maximum temperature of warmest month (Bio05), mean temperature of warmest quarter (Bio10), temperature annual range (Bio07), and temperature seasonality (Bio04), and the aridity index (AI) were also positively correlated with climate PC1, but with a smaller contribution than some of precipitation variables. Potential evapotranspiration (PET), AI, and all the temperature variables except Bio05 and mean temperature of wettest quarter (Bio08) significantly contributed to climate PC2 (*P*<0.01). Precipitation of wettest month (Bio13), annual precipitation (Bio12), precipitation of warmest quarter (Bio18), and precipitation of wettest quarter (Bio16) were negatively correlated with PC2 (*P*<0.05), but the contributions were smaller than those of the temperature variables. To complement the PCA results, we used Spearman correlations to further estimate the interdependence of 19 climate variables, AI, and PET (Supplementary Fig. S8). Two variables for precipitation (Bio12 and Bio15) and six for temperature (Bio01, annual mean temperature; Bio02, mean diurnal range; Bio04; Bio05; Bio08, mean temperature of wettest quarter; and Bio09, mean temperature of driest quarter) were identified. Two significant PCs were detected for soil variables, explaining 43.1 and 21.7% of the variation in the PCA (Fig. 4E). With the exception of C/N ratio (CNrt) and exchangeable sodium percentage (ESP), all soil variables loaded strongly on soil PC1. Among them, only sand content (SDTO) and bulk density (BULK) were negatively correlated with soil PC1. For soil PC2, CNrt and ESP were the most strongly loaded variables. The pH in water (PHAQ) and TEB were positively correlated with soil PC2 and the organic carbon content (ORGC) and total nitrogen content (TOTN) were negatively correlated (*P*<0.001). After accounting for the pairwise correlation coefficients (Supplementary Fig. S9), nine soil variables were isolated: coarse fragments (CFRAG), SDTO, BULK, CNrt, cation exchange capacity of the clay fraction (CECc), TEB, ESP, PHAQ, and calcium carbonate content (CARB). Similar selection processes were carried out for the collection-year weather variables (Fig. 4F; Supplementary Fig. S10). Since PPTtotal and PPTmax were highly correlated with Bio12, and TMP5nb correlated with Bio01, only PPTmin and PPTcv were included as important variables. Thus, a total of 19 variables that described the variabilities of climate, soil, and collection-year weather were selected to estimate their effects on seed size variation among the sand rice populations (Supplementary Fig. S11).

**Fig. 5.**
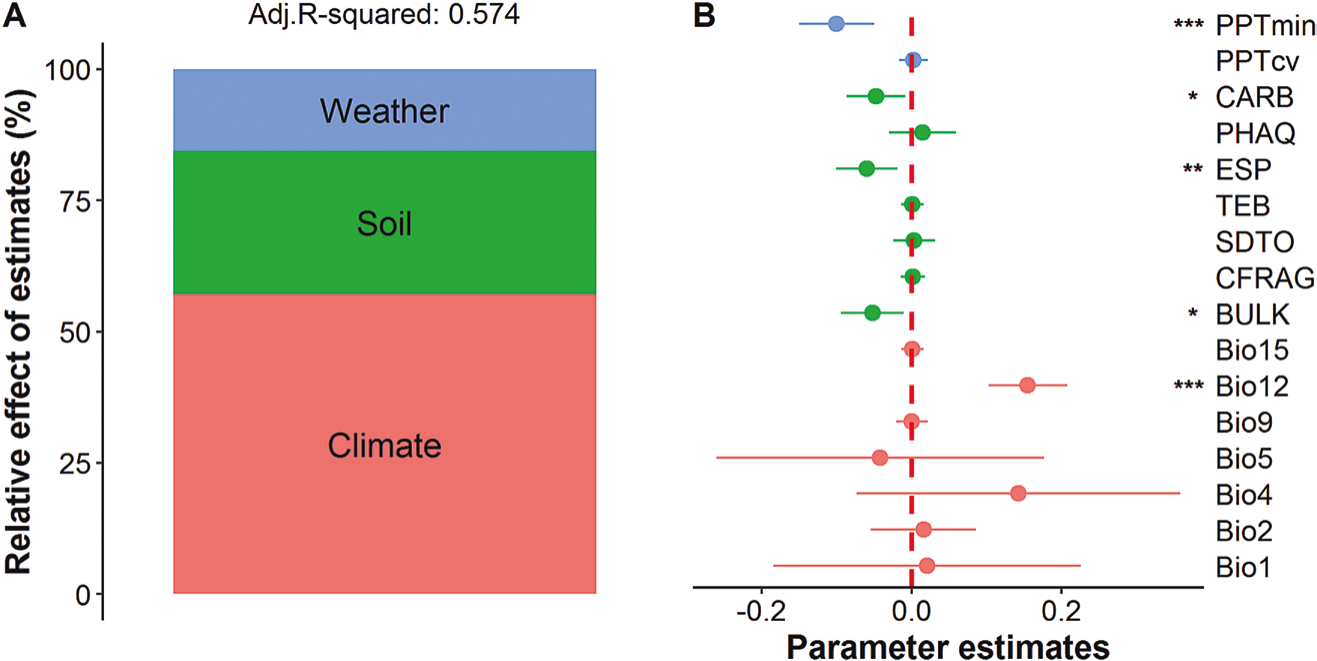
Relative effects of multiple predictors on seed major axis length of sand rice at the population level. (A) Multiple factors are grouped into three components, namely weather, soil, and climate, and the relative importance of each one is expressed as the proportion of the explained variance. The adjusted (adj.) *R*-squared of the average model is shown. (B) The relative effect of each predictor is shown as the mean parameter estimate (standardized regression coefficient), together with its associated 95% confidence interval and *P*-value: *P<0.05; **P<0.01; ***P<0.001. Bio1, annual mean temperature; Bio2, mean diurnal range; Bio4, temperature seasonality; Bio5, max temperature of warmest month; Bio9, mean temperature of driest quarter; Bio12, annual precipitation; Bio15, precipitation seasonality; BULK, soil bulk density; CFRAG, coarse fragments; SDTO, sand content; TEB, total exchangeable bases; ESP, exchangeable sodium percentage; PHAQ, pH in water; CARB, calcium carbonate content; PPTcv, coefficient of variation of monthly precipitation; PPTmin, minimum monthly precipitation.

**Fig. 6.**
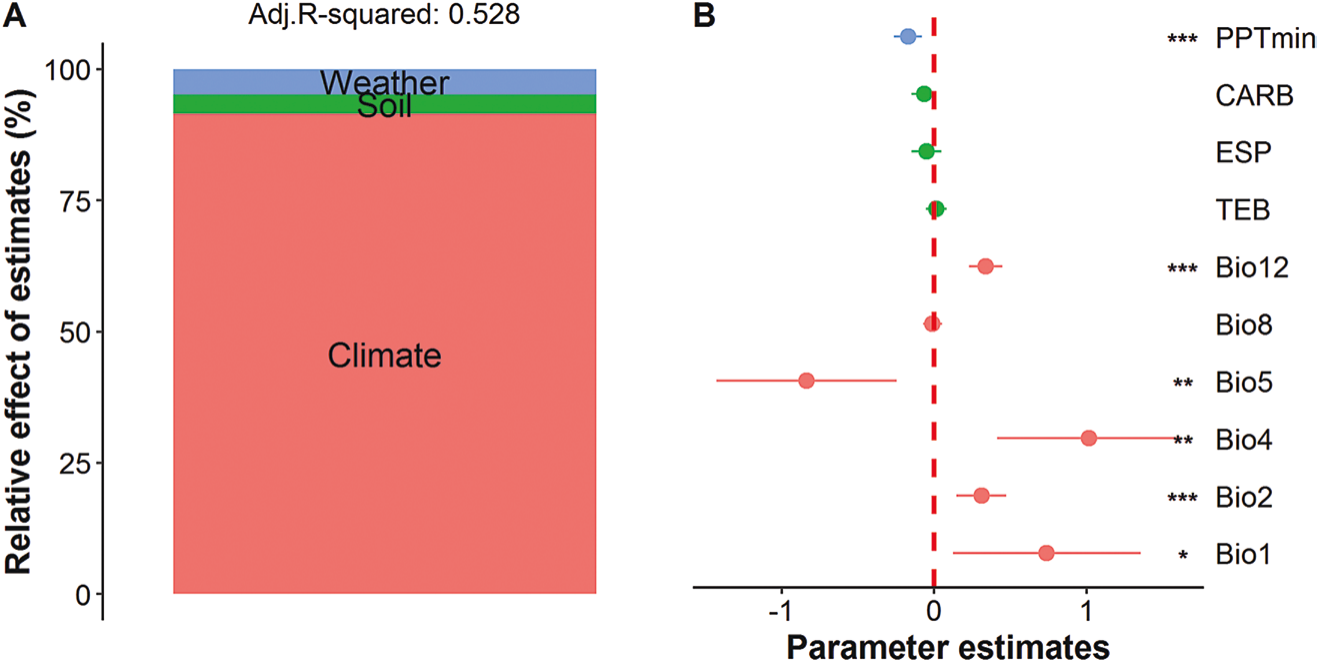
Relative effects of multiple predictors on thousand seed weight of sand rice at the population level. (A) Multiple factors are grouped into three components, namely weather, soil, and climate, and the relative importance of each one is expressed as the proportion of the explained variance. The adjusted (adj.) *R*-squared of the average model is shown. (B) The relative effect of each predictor is shown as the mean parameter estimate (standardized regression coefficient), together with its associated 95% confidence interval and *P*-value: *P<0.05; **P<0.01; ***P<0.001. Bio8, mean temperature of wettest quarter; the other abbreviations are as listed in Fig. 5.

Multiple regression models including all the climate, soil, and collection-year weather predictors explained 57% and 53% (adjusted *R*^2^) of the total variances observed in major axis length and TSW, respectively (Figs 5, 6). Among them, climate variables were the predominant drivers, accounting for 57.20% and 91.54% of the explained variances in major axis length and TSW, respectively. Soil variables explained a high portion of the variance in major axis length (27.27%), but only accounted for 3.64% of the variance in TSW. Similarly, collection-year weather predictors accounted for 15.53% and 4.81% of the variances in major axis length and TSW, respectively. Detailed analyses showed that Bio12 had a significantly positive effect on major axis length while PPTmin had a negative effect (*P*<0.001). The soil variables ESP, CARB, and BULK also had a negative impact on major axis length but to a lesser extent. Unexpectedly, no direct effects were observed for temperature variables on major axis length. For TSW, positive effects were found for Bio01, Bio02, Bio04, Bio05, and Bio12. PPTmin showed a significantly negative effect upon TSW variance; however, soil variables did not impact on TSW.

### Seed size effects on plant performance in a common garden experiment

**Fig. 7.**
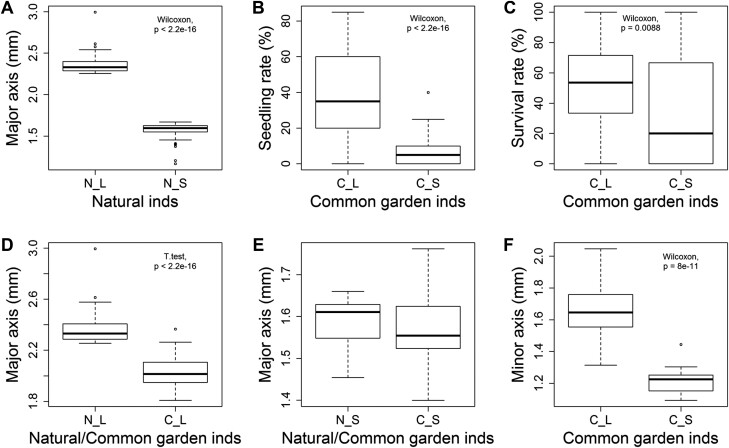
Comparison of characteristics of two contrasting groups with either large (L) or small (S) seeds growing in either natural (N) populations of sand rice or a common garden experiment (C). (A) Major axis length of individual seeds from the natural–large (N_L) and natural–small groups (N_S). (B) Seedling emergence rates of the large and small seeds when grown in the common garden (C_L and C_S, respectively). Emergence was checked continuously and reached its final value at 37 d after sowing. For (A, B) *n*=96. (C) Seedling survival rates for the individuals in the C_L (*n* = 92) and C_S (*n* = 51) groups. Survival was determined at 83 d after sowing. (D) Seed major axis lengths compared between natural and common garden individuals of the large-seed group (N_L versus C_L; *n* = 76). (E) Seed major axis lengths compared between natural and common garden individuals of the small-seed group (N_S versus C_S; *n* = 18). (F) Seed minor axis lengths of the C_L (*n* = 76) and C_S (*n* = 18) individuals. Pairwise statistical tests were performed as indicated in each graph and the *P*-value is given.

**Fig. 8.**
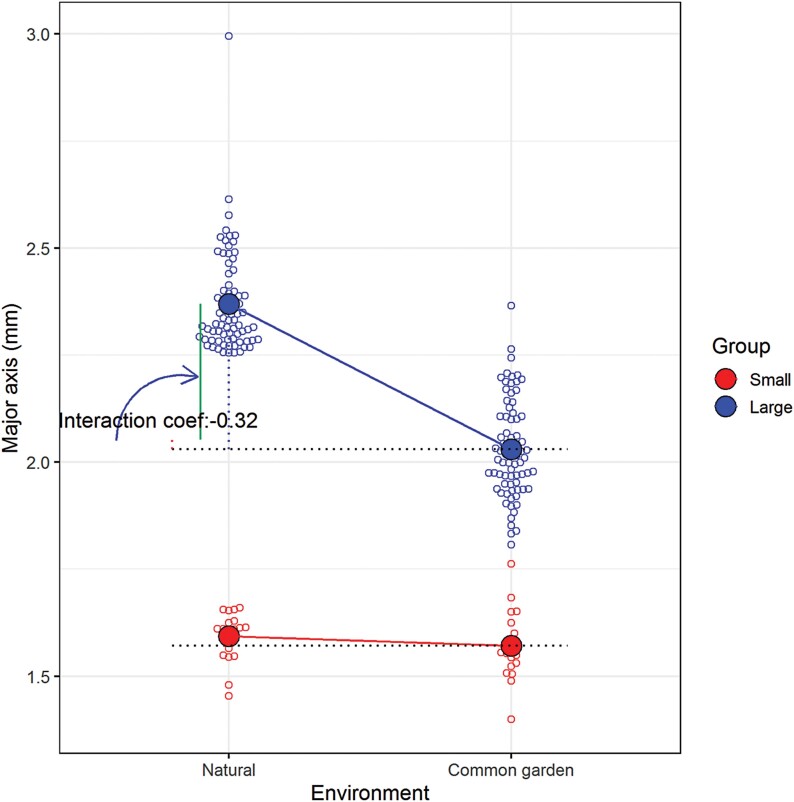
Phenotypic plasticity of seed major axis length of two contrasting groups with either large (L) or small (S) seeds growing in either natural (N) populations of sand rice or a common garden experiment (C). Multiple regressions were conducted to assess the effects of environment (N versus C) and group (L versus S). The interaction coefficient of environment × group is indicated.

To evaluate the effects of seed size variation on functional traits during plant growth, individuals with the largest and the smallest values of major axis length were selected from the natural populations (16 populations for large versus 30 for small) (Supplementary Fig. S12) and each group (N_L, natural-large; and N_S, natural-small) contained 96 independent individual lines. The mean major axis length of the N_L group was 2.37 mm, which was significantly greater than that of the N_S group (1.57 mm; Fig. 7A). Seeds from each individual in the two groups were directly planted into soil in a common garden experiment (hereafter named as C_L, common-large; and C_S, common-small). The seedling emergence rates of C_L group ranged from 0–85%, whereas in C_S group it ranged from 0–40% and seeds from 45 lines did not germinate (Fig. 7B). At 83 d after sowing, of the 92 C_L lines that germinated, seedlings from 11 lines had all died and the overall survival rate was 51.85% (Fig. 7C). The survival rate of C_S lines was significantly lower (36.55%, *P*=0.0088) and seedlings from only 28 of 51 lines survived. The mature plant height of the C_L group was also higher than that of the C_S group (*P*=0.039), partly due to earlier seedling emergence in the C_L group (Supplementary Fig. S13). Consistent with the results in natural populations, seeds harvested from the C_L lines were substantially larger than those from the C_S lines in terms of major and minor axis lengths (*P*<0.001; Fig. 7D–F). When compared with the corresponding lines from natural populations, no significant differences in major axis length were observed in the common garden experiment for the S groups (Fig. 7E), whereas in the L lines the mean length of the major axis was markedly lower in the common garden experiment (Fig. 7D). A multiple regression model was fitted in order to dissect the influence of environment and group on seed size variation, and it showed that the estimated coefficients of intercept, environment, group, and environment × group were 1.59, –0.022, 0.78, and –0.32, respectively (Fig. 8). Further statistical analyses revealed that group and environment × group were significant variables with extremely low *P*-values (*P*=9.95 × 10^–67^ and *P*=2.54 × 10^–13^, respectively). The phenotype of seed size was stable for the S group lines in the common garden experiment when compared with the corresponding natural lines, whereas for the L group lines, the environment × group ­interaction had a significantly negative effect on the major axis length, indicating that the lines with large seeds had stronger plasticity responses to environmental heterogeneity.

## Discussion

### Interspecific versus intraspecific geographic patterns

Comparisons of communities and/or species across distribution gradients have considerably advanced our understanding of the roles of geography and climate on ecosystem functions and life-history trait variations (Moles and Westoby, 2003; Moles *et al*., 2007, 2014; De Frenne *et al*., 2010, 2013). Our study focused on sand rice, which is a widely distributed species in temperate deserts, with the aim of determining the responses of its reproduction traits to complex environmental variables, and we found a longitudinal pattern (west-to-east) of seed size variation. Many interspecific studies have supported the assertion that seed size decreases with increasing latitude (Moles and Westoby, 2003; Moles *et al*., 2007, 2009a; Pakeman *et al.*, 2008; Gallagher and Leishman, 2012; Swenson *et al.*, 2012). Global cross-species analyses have shown that mean seed mass declines seven-fold for every 20° latitude shift towards the poles (Moles and Westoby, 2003), but with an abrupt decrease at the edge of the tropics (Moles *et al.*, 2007). However, a wide range of variability in seed mass exists among different species at the within-site level (De Frenne *et al.*, 2011; Moles *et al.*, 2014; Šímová *et al.*, 2018). Plant growth form, species abundance within the vegetation, and dispersal syndromes are often strongly associated with seed size divergences (Moles *et al*., 2005a, 2005b, 2007, 2009b; Leslie *et al.*, 2017; Šímová *et al.*, 2018). Importantly, the latitudinal cline within shrubs and herbs becomes very weak and the slope coefficient is less than one-tenth that of the cross-species slope (Moles *et al.*, 2007). The vegetation types that have been examined in large-scale interspecific analyses are mostly from forest, grassland, mountain, highland, and warm desert/semi-desert ecosystems (Moles *et al*., 2007, 2014; Pakeman *et al.*, 2008), and there are few studies of the temperate desert in Central Asia. In our study, we applied intraspecific comparisons in order to examine the environmental variables associated with seed size variation in a pioneer species in temperate deserts (Fig. 1).

As with the interspecific patterns, some studies have also reported a negative latitudinal pattern for intraspecific seed size variation (Moles and Westoby, 2003; De Frenne *et al.*, 2013); however, the relationship was extremely weak and the slope coefficient was only ~29% of that of interspecies analyses (Moles and Westoby, 2003). Examination of the species presented in Moles and Westoby (2003) shows that 36 and two species displayed negative and positive latitudinal patterns, respectively, but 41 species exhibited no significant relationship between latitude and seed mass. Similarly, data in De Frenne *et al.* (2013) indicate that there were 24 and six species respectively showing a decline and increase in seed size with latitude of origin, whilst 33 species showed non-significant latitudinal clines. In addition, latitudinal clines have also been found to be absent in some annual and forest herbs (Murray *et al.*, 2004; De Frenne *et al*., 2009, 2010, 2011; Soper Gorden *et al.*, 2016). Our results also showed that latitude was not a predominate variable driving the variation in seed size across natural populations sand rice (Fig. 3D, E). Hence, whereas negative latitudinal clines are observed in cross-species studies, within species their presence appears to be species-specific rather than constant.

Moles and Westoby (2003) reported that for species with a latitudinal range of more than 11° there is a high probability of finding a significant decline in seed size along latitude. Yin *et al.* (2016b) have previously found a positive relationship between seed size and latitude across 26 sand rice populations that had latitudinal and longitudinal ranges of 36.17–43.71° N and 94.65°–122.84° E, respectively. However, seed size variation was found to be positively correlated with latitude and longitude when 16 of the 26 populations were analysed using redundancy analysis and Pearson correlation analysis (same range of latitude; longitude 98.12–122.84° E; Yin *et al.*, 2016a). One factor possibly underlying these geographic patterns is that the study ranges were located in a climate transition zone. For example, Yin *et al.* (2016b) studied 10 populations from the Horqin and Otindag sand fields in a semi-arid region whilst the other 16 populations were from deserts in arid regions in China, and the equivalent numbers for Yin *et al.* (2016a) were five and 11 populations. The sampled latitudinal and longitudinal ranges of our study were 20.24° (29.24–49.48° N) and 62.96° (59.88–122.84° E), respectively (Fig. 1), representing most of the documented distribution range and the entirety of precipitation gradients of the range of sand rice. Our study range was wider compared to previous sand rice studies and also wider compared with most studies of other species (De Frenne *et al.*, 2013; Moles and Westoby, 2003). Unlike previous observations from common garden experiments, which mainly reflect local adaptation and genetic differentiation, quantification of natural populations of sand rice might more clearly reveal the natural selection and phenotype plasticity that occurs in response to environment, and will present a broader perspective on patterns of seed size variation across large geographic gradients. In our case, bivariate linear models indicated that longitude had significant and positive effects on major axis length and TSW (Fig. 3), but latitudinal patterns were not found. Hierarchical cluster analysis based on climatic variables categorized our 68 populations into seven groups (Fig. 3A), which were well matched with the extreme arid, arid, and semi-arid regions from west to east in Northern China. These results suggest that geographic variation in the seed size of sand rice might be largely shaped by climatic variables.

### Climatic and local environmental drivers of seed size variation

The major axis length and TSW varied extensively with ~1.90-fold and 5.95-fold changes, respectively, across the natural populations of sand rice (Supplementary Fig. S5). The lowest values came from a population in Gurbantunggut in an extreme arid region in Xinjiang, northwest China. Multiple regression models found 16 and 10 ten potential variables contributed to the observed variances of major axis length and TSW, respectively (Figs 5, 6). The results of the best model selections were also validated using the ‘*step()*’ function implemented in R and the *leaps* R package (Supplementary Fig. S14). The long-term climate variables accounted for most of the explained variances of major axis length and TSW. Specifically, annual precipitation (Bio12) had the most significantly positive effects on seed size, suggesting that the longitudinal clines are driven by a precipitation gradient from west to east across the sand rice distribution range. Previous large-scale interspecific analyses have hypothesized that mean annual temperature is a strong predictor of seed size variation (De Frenne *et al.*, 2009; Murray *et al.*, 2004; Pakeman *et al.*, 2008; Moles *et al.*, 2014). In our study, annual mean temperature (Bio01), temperature seasonality (Bio04), and mean diurnal range (Bio02) had pronounced and positive effects on TSW, while maximum temperature of warmest month (Bio05) had a negative effect, which generally confirmed the previous cross-species patterns, but only a small and non-significant effect of temperature was observed for major axis length. These discrepancies in the response could partly result from the fact that the final sizes of seeds and grains are mainly determined by the growth of the integuments in dicot plants (e.g. Arabidopsis) and by the spikelet hull in monocot plants (e.g. *Oryza sativa*; Li *et al.*, 2019), respectively, while seed filling substantially contributes to seed weight (Wang *et al.*, 2008). Importantly, seed filling is very sensitive to heat stress and temperature-mediated drought stress (Sehgal *et al.*, 2018). The fact that mean annual ­precipitation is a poor indicator of water availability for plants at global level leads to it being a weak explanatory variable for seed mass (Moles *et al.*, 2014). However, mobile sand dunes in Central Asia differ considerably from the ecosystems included in global cross-species analyses, as the mean annual precipitation is highly correlated with the precipitation during the growth season. Furthermore, sand rice is a pioneer species and does not have a deep root system, instead possessing advanced lateral roots to access the available surface water (Chen *et al.*, 2014). Sand rice is also a heat-tolerant herb that can withstand heat treatments of >50 °C (Zhao *et al.*, 2014). In addition, we found that minimum monthly precipitation (PPTmin) had a marked negative effect on major axis length and TSW (Figs 5, 6), and was consistent with previous cross-species patterns that the seed mass of annual herbs co-varies with the collection-year weather (Soper Gorden *et al.*, 2016). Overall, our findings demonstrated that precipitation has a strong explanatory power on seed size variation; however, it is important to note that our results do not cast doubt upon previous global interspecific studies of seed mass owing to the relatively small range that we used and the fact that we only studied a single species.

Soil characteristics and fertility strongly affect the performance of plants. Global interspecific analyses have shown that soil variables, especially pH and available phosphorus, are major predictors of leaf photosynthetic traits and rates (Maire *et al.*, 2015), but the effects of soil variables on seed mass have been found to be species-dependent among some forest herbs (De Frenne *et al.*, 2009). Sand rice grows in extremely nutrient-poor environments and most of our sampling sites had values of total exchangeable bases of <65 (Fig. 4B and Supplementary Table S1). Substantial effects of soil variables were found for major axis length, whereas they only explained a small portion of TSW variation (Figs 5, 6), suggesting that that axis length and TSW respond differently to local environmental variables. In addition, the soil texture variable bulk density and the ion-exchange capacity variables exchangeable sodium percentage and calcium carbonate content negatively influenced the variation in major axis length. Taken together, our findings highlight the importance of climatic, collection-year weather, and soil variables in concurrently driving the longitudinal variation in seed size. However, other unaccounted factors, such as photoperiod, wind, and available phosphorus, might also be associated with seed size variation, hence further multi-factorial analyses with more available environmental factors are required to advance our knowledge of the specific ecological processes that underly the geographic variation in seed size of sand rice.

### Phenotype plasticity, local adaptation, and domestication

Due to its wide distribution, exceptional nutritional value, and high stress tolerance, sand rice has been promoted as a potential climate-resilient crop (Chen *et al.*, 2014; Zhao *et al.*, 2021). Seed size is a key domestication trait in addition to its important ­ecological function. High phenotypic variation among sand rice populations has been observed in previous studies as well as in our current one (Yin *et al*., 2016a, b; Zhang *et al.*, 2018). Intraspecific variation is a consequence of the combination of genetic variation, phenotypic plasticity to environmental changes, and genotype × environment interactions (Ren *et al.*, 2020; Anderegg *et al.*, 2021). Hence, studying the heritable variation, plastic capacity, and fitness of seed size is critical for predicting the responses of sand rice to complex desert environments, and is an important step towards achieving successful domestication. Common garden experiments are ideal for assess underlying ecological processes of within-species variation (Ren *et al.*, 2020). Previous studies have documented genetic differentiation in seed size within limited populations and geographical areas (Yin *et al*., 2016a, b;), but a broader picture of the ecological mechanisms underlying seed size variation is still lacking. In our study, we selected two contrasting groups in terms of seed size to perform a common garden experiment, namely large seeds (L) and small seeds (S). Interestingly, most of the large individuals from natural populations (N_L) came from the Hulun Buir, Horqin, and Otindag sand fields in the semi-arid region in north-east China, whereas the corresponding small individuals (N_S) were from deserts in the extreme arid and arid regions of China and Kazakhstan (Supplementary Fig. S12). This suggests that individuals with large seeds are more competitive in moist environments, especially in the semi-arid sand fields. Comparisons of the individuals in the large group between the natural populations (N_L) and the common garden (C_L) showed that the latter were highly plastic for seed size (Fig. 7D). The pronounced decline in seed size in the C_L individuals might have been the result of the different climatic conditions in the common garden compared to the geographic origins of the N_L individuals. In contrast, the mean seed size of the small group was only slightly reduced in the common garden when compared with the corresponding individuals from the natural populations (Fig. 7E). A plausible explanation is that the common garden site was located in an arid region (Lanzhou), and the possibility that the capacity for phenotypic changes is more constrained in extreme arid and arid environments cannot be ruled out. Furthermore, the mean seed size of the C_L group was significantly higher than that of the C_S group for the lengths of both the major and minor axes in common garden (Fig. 7D–F), indicating that the plasticity of seed size is heritable and potentially adaptive. Population genetic analyses have consistently indicated that genetic divergence mainly occurs between populations from the eastern sand fields and those from the central and western deserts in Northern China (Qian *et al*., 2016, 2021). Our previous haplotype analysis of a seed size candidate gene, *DA1-Related*, also showed a similar divergence pattern among natural populations of sand rice (Zhao *et al.*, 2017). Overall, the adaptive plasticity of seed size from the north-eastern populations would provide a potential advantage to cope with climate change.

Many advantages of large seeds have been determined with respect to dormancy, germination, seedling size, and seedling survival at early stages of growth (Moles, 2018). For example, larger seeds have less dormancy and generate larger seedlings with higher survival rates (Lloret *et al.*, 1999; Moles and Westoby, 2004; Benard and Toft, 2007; Larios *et al.*, 2014; Rubio de Casas *et al.*, 2017; Moles, 2018). Seedlings from large seeds can emerge successfully from deep litter, from greater depths in the soil, and from deeper sand burial (Westoby *et al.*, 1992; Li *et al.*, 2006). An increased seed size is also assumed to prompt seedling emergence from deeper burial within tilled fields, and is one of the most documented syndromes during the process of crop domestication (Fuller *et al.*, 2014). In sand rice natural habitats, seasonal precipitation and soil temperature drive the cycling of dormancy in the soil seed bank (Gao *et al.*, 2018), but some contrasting results for physiological dormancy have been reported in freshly harvested seeds (Wang *et al.*, 1998; Fan *et al*., 2016, 2017). Sand rice germination exhibits a unimodal continuous pattern, reaching its highest rate at 7 d after sowing in growth-chamber conditions, and it is highly sensitive to the light and temperature conditions (Wang *et al.*, 1998; Zheng *et al.*, 2004). In our common garden experiment, the seedling emergence and survival rates were significantly lower in C_S group than in the C_L group (Fig. 7B, C), which supports previously reported interspecific relationships between seed size and seedling emergence and establishment (Moles, 2018), and is also consistent with our agronomic assessment in loess soils (Zhang *et al.*, 2018). However, inherent differences in seed ageing, dormancy, and vigor between the two seed size groups might also account for the lower rates of seedling emergence and survival in the C_S group. Synchronous seedling emergence and high survival rates are prerequisites for domestication of new crops.

Interestingly, our previous field experiments have shown that individuals from semi-arid regions (e.g. Duolun, Naiman, and Aerxiang) exhibit compact architecture, early flowering time, and have favorable harvest indices (Zhang *et al.*, 2018). The yield of the genotype from Aerxiang in loess soil is estimated to reach up to 129.55 g m^–2^, which compares well with quinoa cultivated in Dubai on the Arabian Peninsula (Choukr-Allah *et al.*, 2016). Overall, the greater adaptive plasticity, higher potential for early seedling establishment, and better agronomical performance of the large-seeded sand rice genotypes from the semi-arid region of northeast China suggest that they provide the best materials for future breeding.

## Supplementary data

The following supplementary data are available at *JXB* online.

Table S1. Primary data for seed size, long-term climate variables, soil variables, and collection-year weather variables for the natural populations.

Fig. S1 Schematic of major and minor axis positions of sand rice seed.

Fig. S2. Linear mixed-effect models of major axis length and TSW according to longitude at the population level.

Fig. S3. Linear models of major axis length according to longitude at the individual level.

Fig. S4. Linear models of TSW according to longitude at the individual level.

Fig. S5. Major axis length and TSW values at the population and individual levels, and frequency distributions.

Fig. S6. Model predictions of sand rice distribution.

Fig. S7. Linear models of major axis length and TSW according to longitude at the population level.

Fig. S8. Correlation analysis among the long-term climatic variables.

Fig. S9. Correlation analysis among the soil variables.

Fig. S10. Correlation analysis among the collection-year weather variables.

Fig. S11. Correlation analysis among the 19 variables selected as being important.

Fig. S12. Geographic distribution of individuals in the two contrasting seed size groups.

Fig. S13. Seedling emergence and plant heights in the common garden experiment.

Fig. S14. Important predictors selected using the *leaps* R package for linear models of major axis length and TSW at the population level.

erac231_suppl_Supplementary_Figures_S1-S4

erac231_suppl_Supplementary_Table_S1

## Data Availability

All data supporting the findings of this study are available within the paper and within its supplementary materials published online.
