# Supplementary material for: Precipitation and local environment shape the geographic variation of seed size across natural populations of sand rice (Agriophyllum squarrosum)
Source: J Exp Bot. 2022 May 23;73(16):5682–97. doi: 10.1093/jxb/erac231 (PMC9467651; doi:10.1093/jxb/erac231)

Supplementary information

Table S1 Primary data of seed size, long-term climate variables, soil variables, collection-year weather variables and the collection year. Name, population names; Individual_number_MA, individual number in each population used for measuring major axis; MA, major axis; Individual_number_TSW, individual number in each population used for measuring thousands of seed weight; TSW, thousands of seed weight; Desert_origin, desert name of each population sampled; Group_Figure 3, group of each population categorized in Figure 3 in main text; Lng, longitude; Lat, latitude; Bio1, annual mean temperature; Bio2, mean diurnal range; Bio3, isothermality; Bio4, temperature seasonality; Bio5, max temperature of warmest month; Bio6, min temperature of coldest month; Bio7, temperature annual range; Bio8, mean temperature of wettest quarter; Bio9, mean temperature of driest quarter; Bio10, mean temperature of warmest quarter; Bio11, mean temperature of coldest quarter; Bio12, annual precipitation; Bio13, precipitation of wettest month; Bio14, precipitation of driest month; Bio15, precipitation seasonality; Bio16, precipitation of wettest quarter; Bio17, precipitation of driest quarter; Bio18, precipitation of warmest quarter; Bio19, precipitation of coldest quarter; PET, potential evapotranspiration; AI, aridity index; CFRAG, coarse fragments; SDTO, sand content; STPC, silt content; CLPC, clay content; BULK, bulk density; TAWC, available water capacity; ORGC, organic carbon content; TOTN, total nitrogen content; CNrt, C/N ration; CECs, cation exchange capacity of fine earth fraction; CECc, cation exchange capacity of clay fraction; TEB, total exchangeable bases; BSAT, base saturation as percentage of CECsoil; ESP, exchangeable Na percentage; PHAQ, pH in water; ELCO, electrical conductivity; CARB, calcium carbonate content; PPTtotal, total precipitation from last October to the October of the collection year; PPTmin, minimum monthly precipitation; PPTmax, maximum monthly precipitation; PPTcv, coefficient of variation of monthly precipitation; TMP5nb, number of days with daily temperature above 5 ℃; Year, the year of each population sampled. The data predicted by the *knnImputation* R package are highlighted in red and bold.

Figure S1 Schematic of major and minor axis positions of sand rice seed. The red line shows the major axis and the blue line shows the minor axis measured in this study.

Figure S2 Major axis and TSW values. (A) Boxplots of major axis values at population and individual levels. (B) Histogram of the average major axis values for 68 populations. (C)

Histogram of the average major axis values for 871 individuals in 68 populations. The green and yellow dash lines show the threshold values of the smallest and largest individuals selected for the following common garden assay. (D) Boxplots of TSW values at population and individual levels. (E) Histogram of the average TSW values for 64 populations. (F) Histogram of the average TSW values for 818 individuals in 68 populations. The measured TSW value of the YJ_YuanZ population was included in D-F. Wilcoxon tests were performed in A and D and the *p* values are shown. The red lines in B, C, E, and F are the fitted normal distribution curves.

Figure S3 The predicted sand rice distribution model results. The predicted results by Mahal, Maxent, and average score of these two models are presented and the boundary line of P. R. China is highlighted in dark red.

Figure S4 Linear model results of major axis and TSW at population levels. Only longitude (Lng) is included as predictor for major axis (A) and TSW (B). These two photos are generated by *ggeffects* R package and the adjusted *R-squared* values are shown at the bottom of each photo.

Figure S5 Linear mixed-effect models of major axis and TSW at population levels. Longitude and latitude are included as predictors in A and C, while only is longitude included in B and D. All photos are generated by *ggeffects* R package and the marginal *R-squared* values are shown at the bottom of each photo.

Figure S6 Linear models of major axis at individual level. Longitude and latitude are included as predictors in A and C, while only is longitude included in B and D. The major axis values were log transformed before model constructions. All photos are generated by *ggeffects* R package and the adjusted *R-squared* values are shown at the bottom of each photo.

Figure S7 Linear models of TSW at individual level. Longitude and latitude are included as predictors in A and C, while only is longitude included in B and D. The TSW values were log transformed before model constructions. All photos are generated by *ggeffects* R package and the adjusted *R-squared* values are shown at the bottom of each photo.

Figure S8 Correlation analysis of the long-term climatic variables. The original data of each variable were used for correlation analysis by *ggcorrplot* R package.

Figure S9 Correlation analysis of the soil variables. The original data of each variable were used for correlation analysis by *ggcorrplot* R package.

Figure S10 Correlation analysis of the collection-year weather variables. The original data of each

variable were used for correlation analysis by *ggcorrplot* R package.

Figure S11 Correlation analysis of 19 important variables. The original data of each variable were z-score transformed before the correlation analysis using the *ggcorrplot* R package. The boundary of P. R. China is shown with the cartographic line.

Figure S12 Geographic distribution of individuals in two extreme groups. The large individuals are shown in squares filled with blue and the small individuals are shown in circles filled with brown. The purple triangle represents that a large individual and a small one are selected from the same population.

Figure S13 Seedling emergence days and plant height in common garden. (A) The days of seedling emergence of the large (C_L) and the small (C_S) group individuals. (B) The final plant height values of the C_L and the C_S group individuals. Statistical tests were performed and the *p* values are shown in each photo.

Figure S14 Important predictors selected by *leaps* R package for linear models of major axis (A) and TSW (B) at population level. The *leaps* function was used for the best subsets regression and all possible combination of the variables for major axis or TSW was tested according to the adjr2.

Supplementary Table

Table S1 Primary data of seed size, long-term climate variables, soil variables, collection-year weather variables and the collection year.

This table is shown as a Excel .xlsx file.

Supplementary Figures

Figure S1 Schematic of major and minor axis positions of sand rice seed. The red line shows the major axis and the blue line shows the minor axis measured in this study.

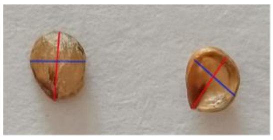

Figure S2 Major axis and TSW values. (A) Boxplots of major axis values at population and individual levels. (B) Histogram of the average major axis values for 68 populations. (C) Histogram of the average major axis values for 871 individuals in 68 populations. The green and yellow dash lines show the threshold values of the smallest and largest individuals selected for the following common garden assay. (D) Boxplots of TSW values at population and individual levels. (E) Histogram of the average TSW values for 64 populations. (F) Histogram of the average TSW values for 818 individuals in 68 populations. The measured TSW value of the YJ_YuanZ population was included in D-F. Wilcoxon tests were performed in A and D and the *p* values are shown. The red lines in B, C, E, and F are the fitted normal distribution curves.

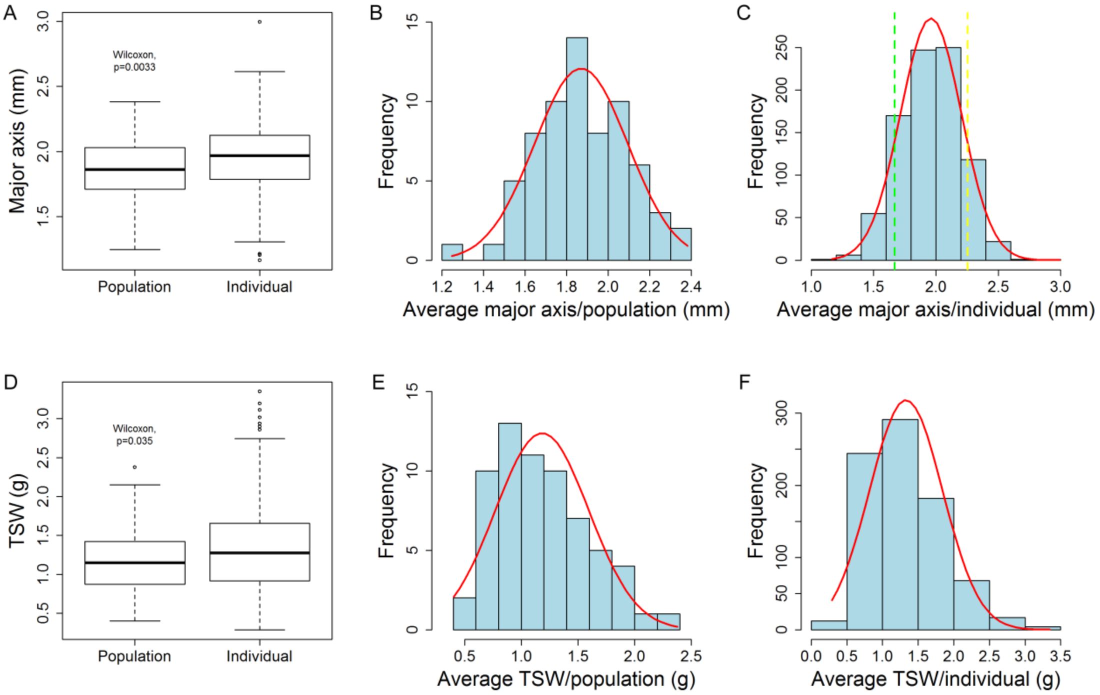

Figure S3 The predicted sand rice distribution model results. The predicted results by Mahal, Maxent, and average score of these two models are presented and the boundary line of P. R. China is highlighted in dark red.

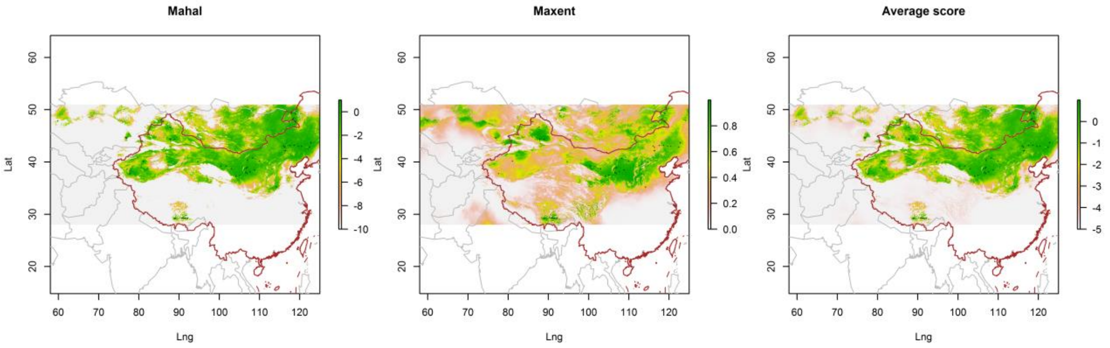

Figure S4 Linear model results of major axis and TSW at population levels. Only longitude (Lng) is included as predictor for major axis (A) and TSW (B). These two photos are generated by *ggeffects* R package and the adjusted *R-squared* values are shown at the bottom of each photo.

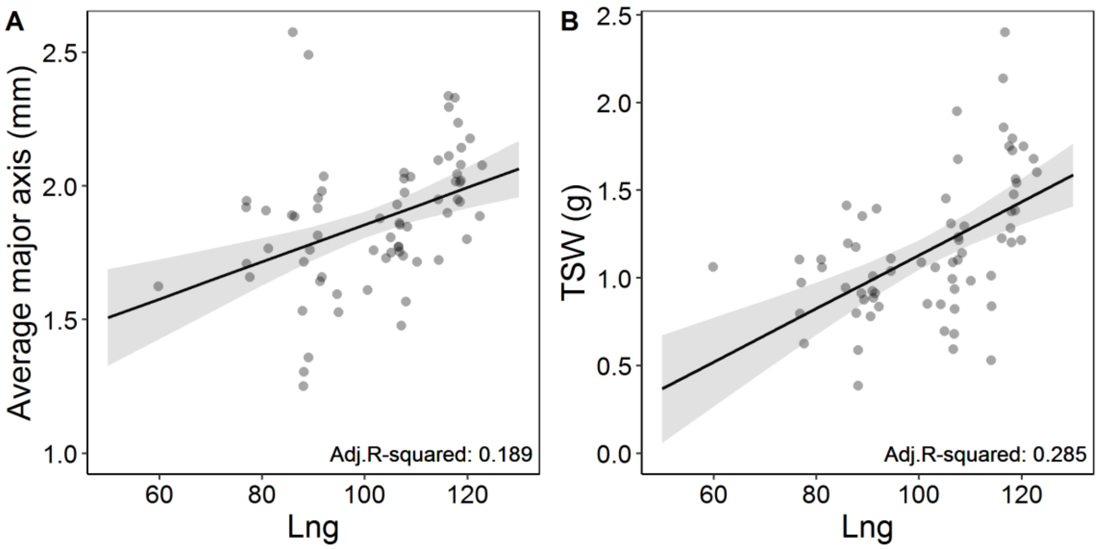

Figure S5 Linear mixed-effect models of major axis and TSW at population levels. Longitude and latitude are included as predictors in A and C, while only is longitude included in B and D. All photos are generated by *ggeffects* R package and the marginal *R-squared* values are shown at the bottom of each photo.

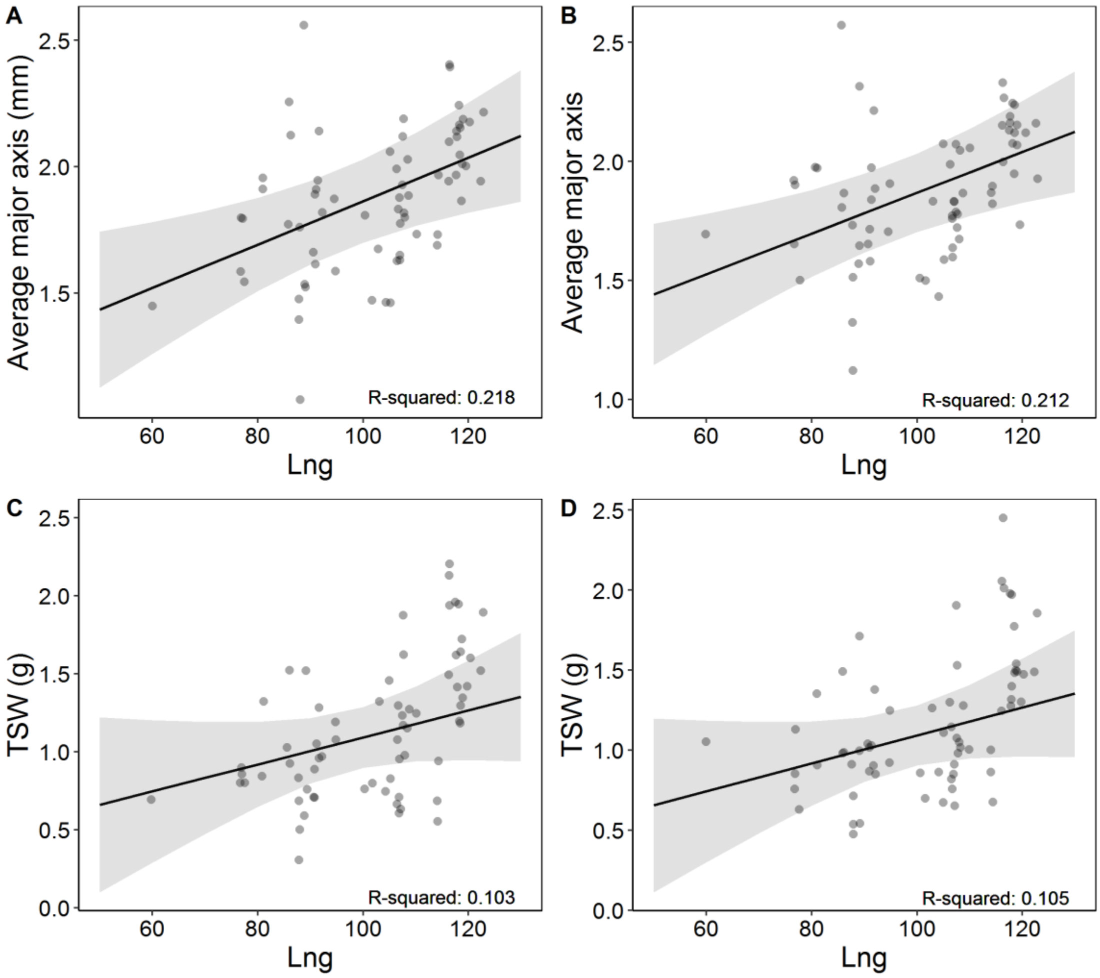

Figure S6 Linear models of major axis at individual level. Longitude and latitude are included as predictors in A and C, while only is longitude included in B and D. The major axis values were log transformed before model constructions. All photos are generated by *ggeffects* R package and the adjusted *R-squared* values are shown at the bottom of each photo.

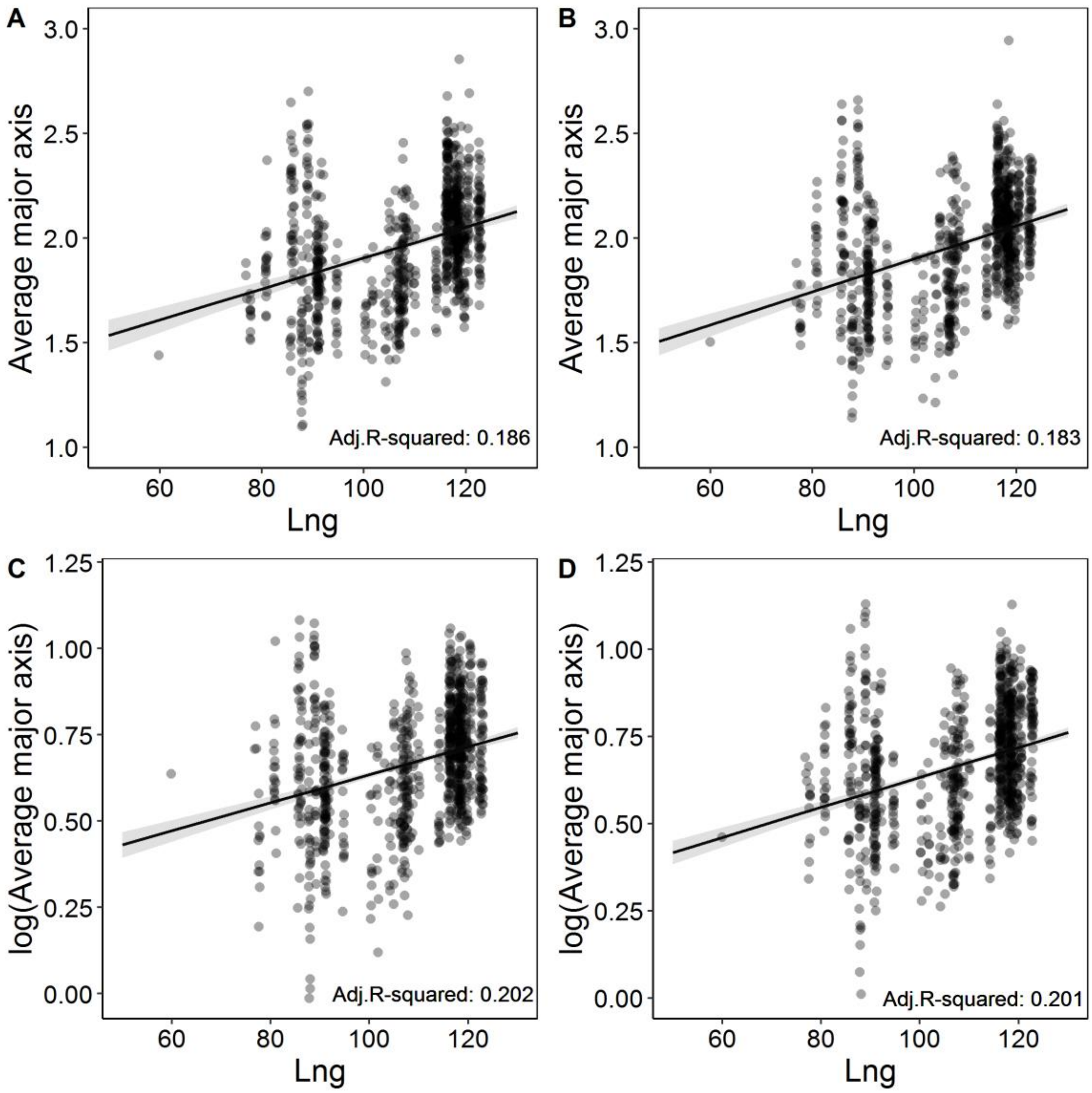

Figure S7 Linear models of TSW at individual level. Longitude and latitude are included as predictors in A and C, while only is longitude included in B and D. The TSW values were log transformed before model constructions. All photos are generated by *ggeffects* R package and the adjusted *R-squared* values are shown at the bottom of each photo.

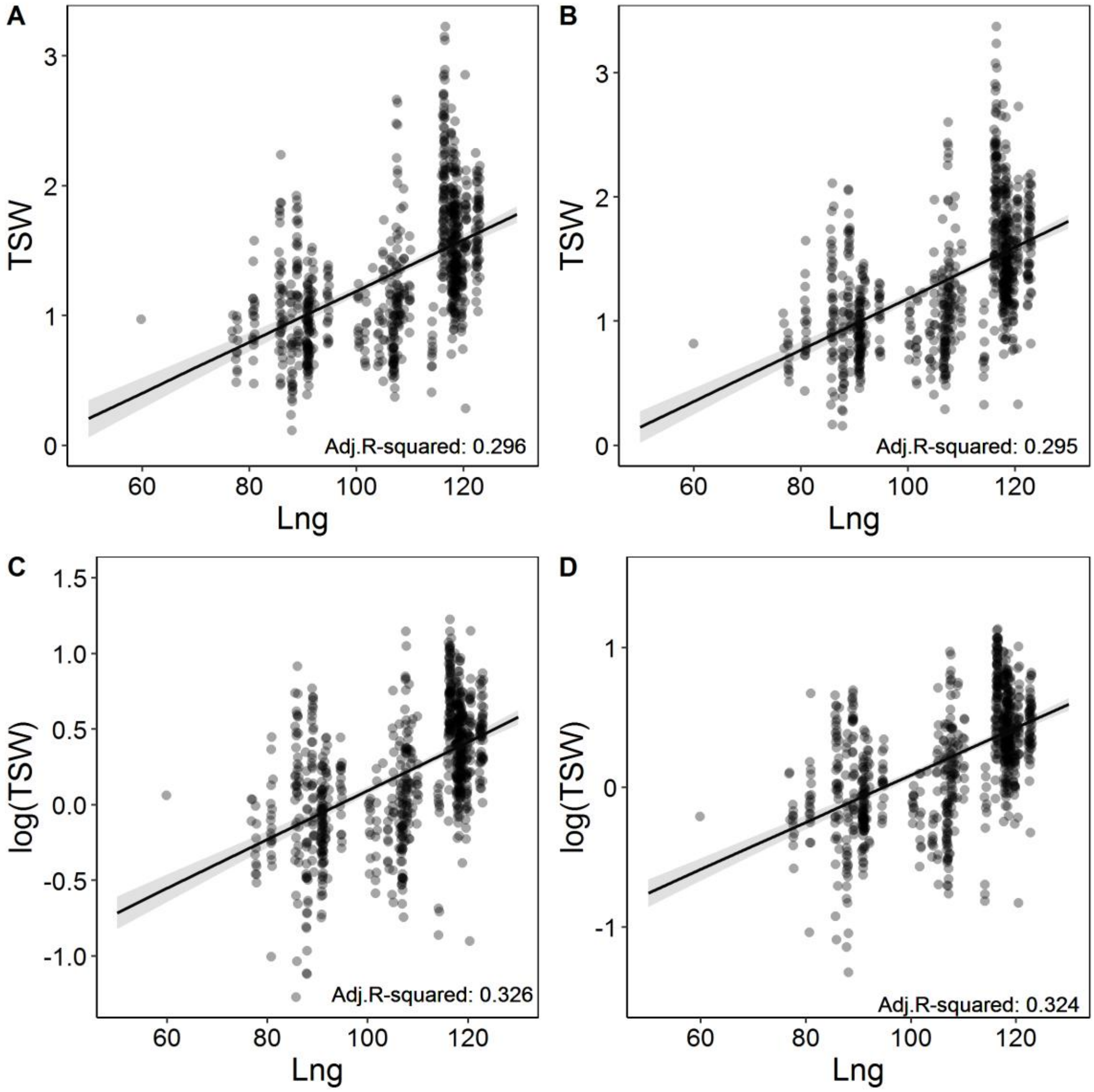

Figure S8 Correlation analysis of the long-term climatic variables. The original data of each variable were used for correlation analysis by *ggcorrplot* R package.

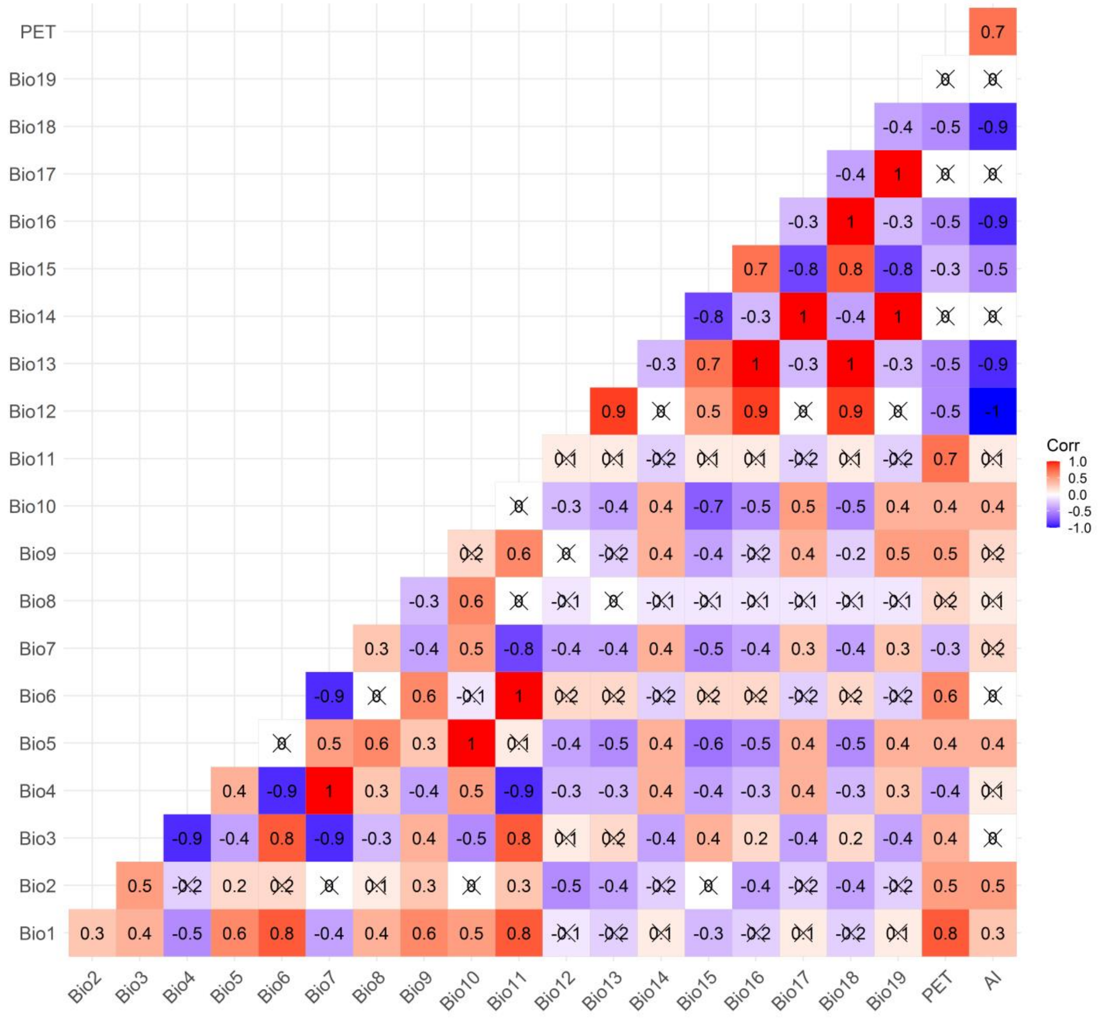

Figure S9 Correlation analysis of the soil variables. The original data of each variable were used for correlation analysis by *ggcorrplot* R package.

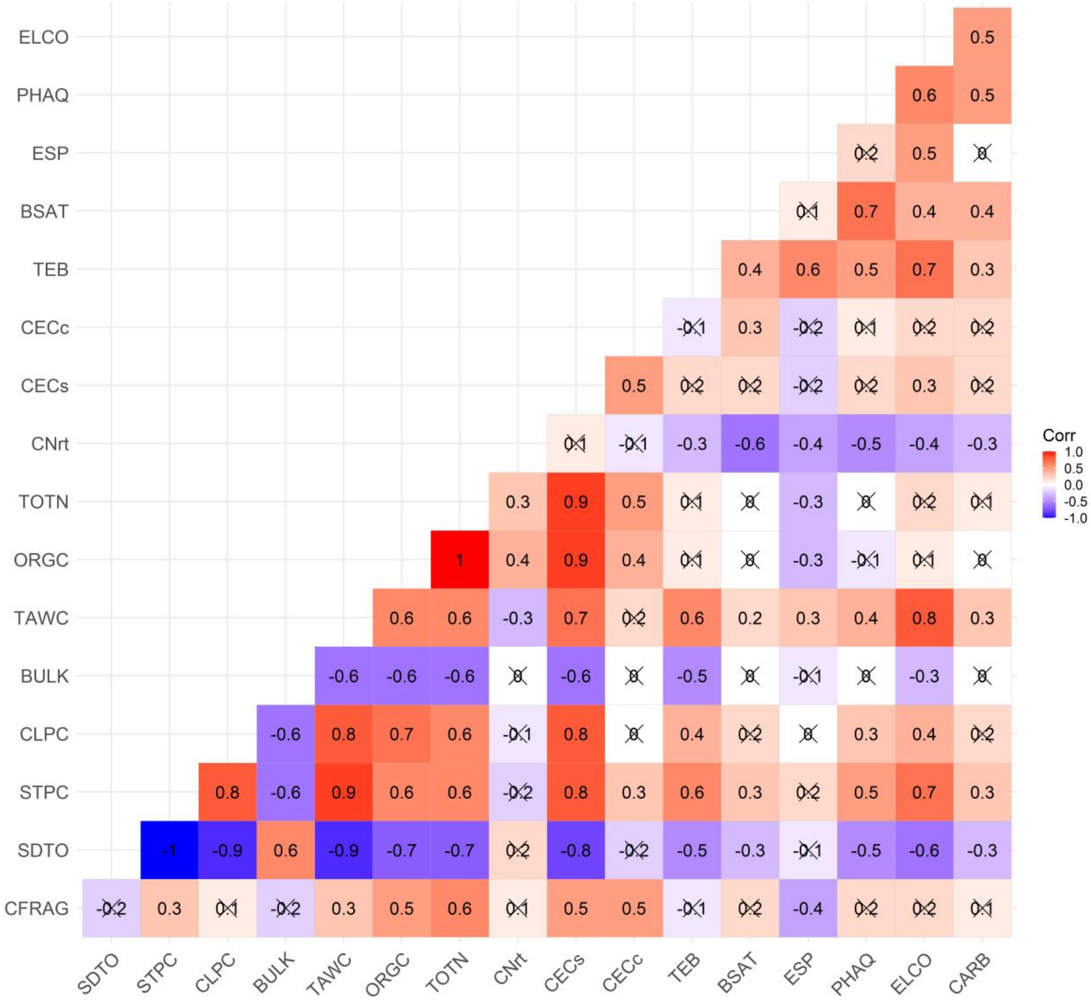

Figure S10 Correlation analysis of the collection-year weather variables. The original data of each variable were used for correlation analysis by *ggcorrplot* R package.

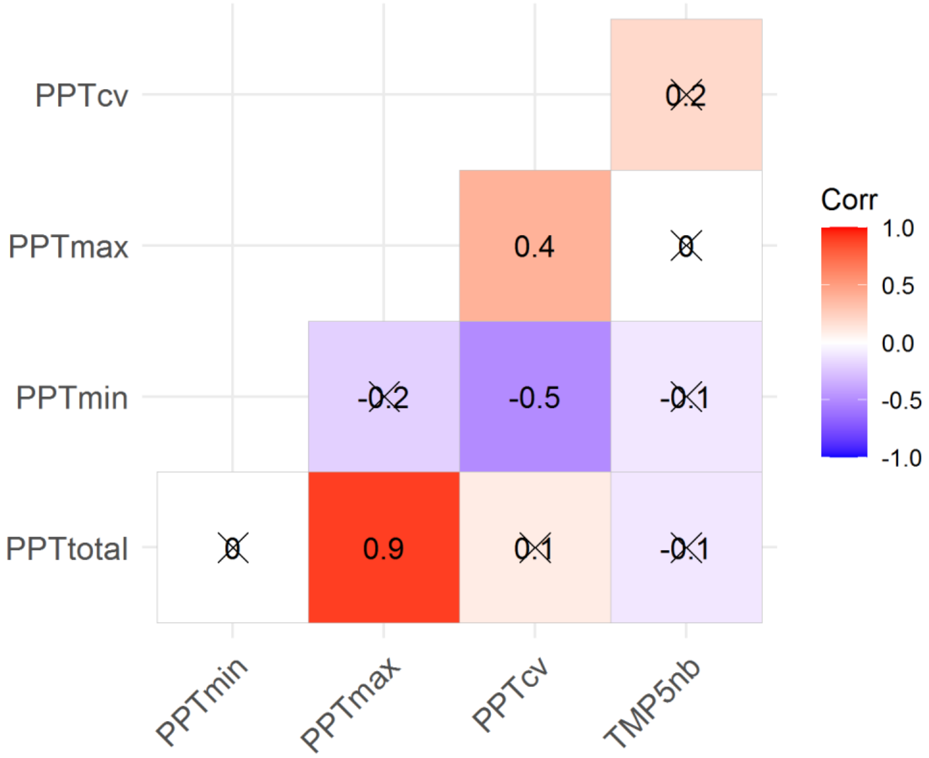

Figure S11 Correlation analysis of 19 important variables. The original data of each variable were z-score transformed before the correlation analysis using the *ggcorrplot* R package. The boundary of P. R. China is shown with the cartographic line.

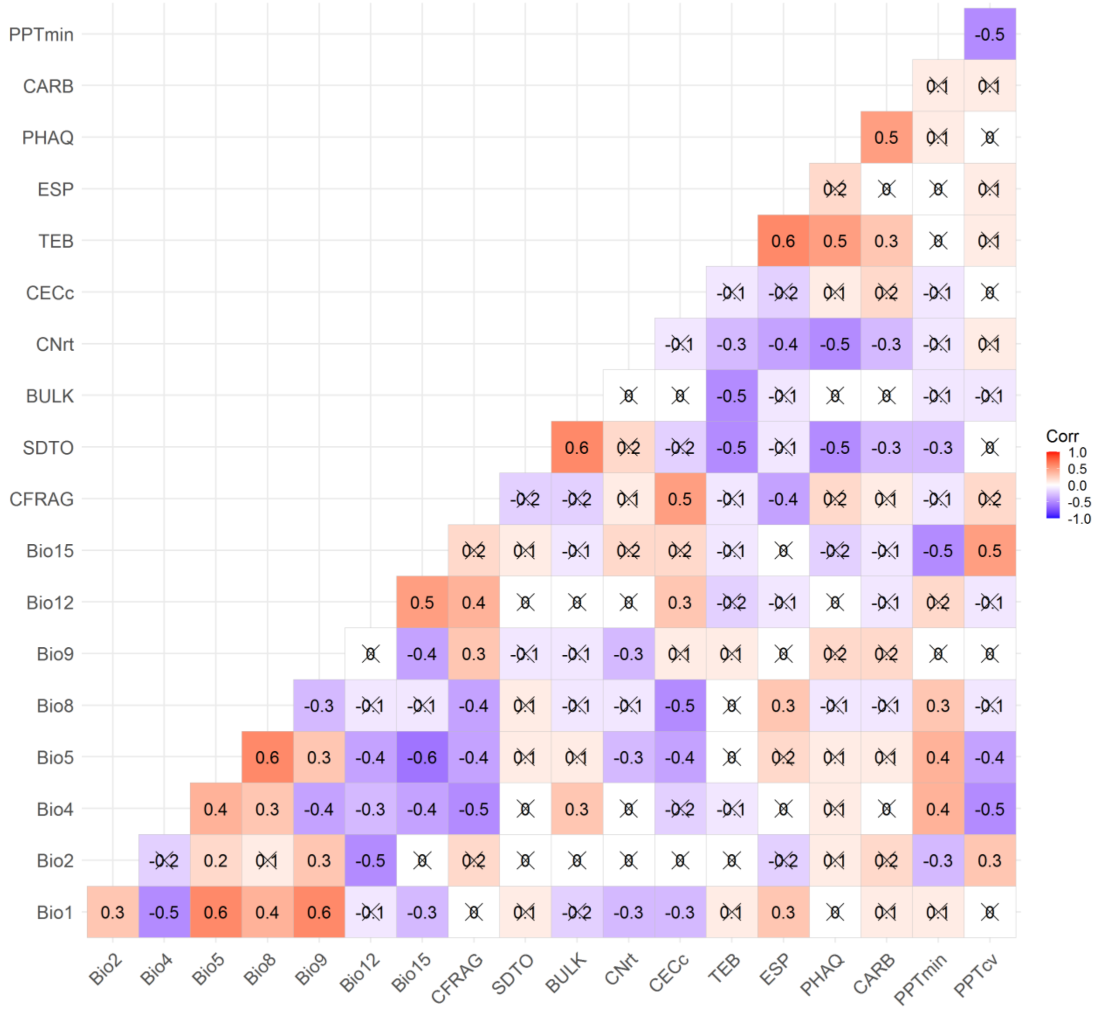

Figure S12 Geographic distribution of individuals in two extreme groups. The large individuals are shown in squares filled with blue and the small individuals are shown in circles filled with brown. The purple triangle represents that a large individual and a small one are selected from the same population.

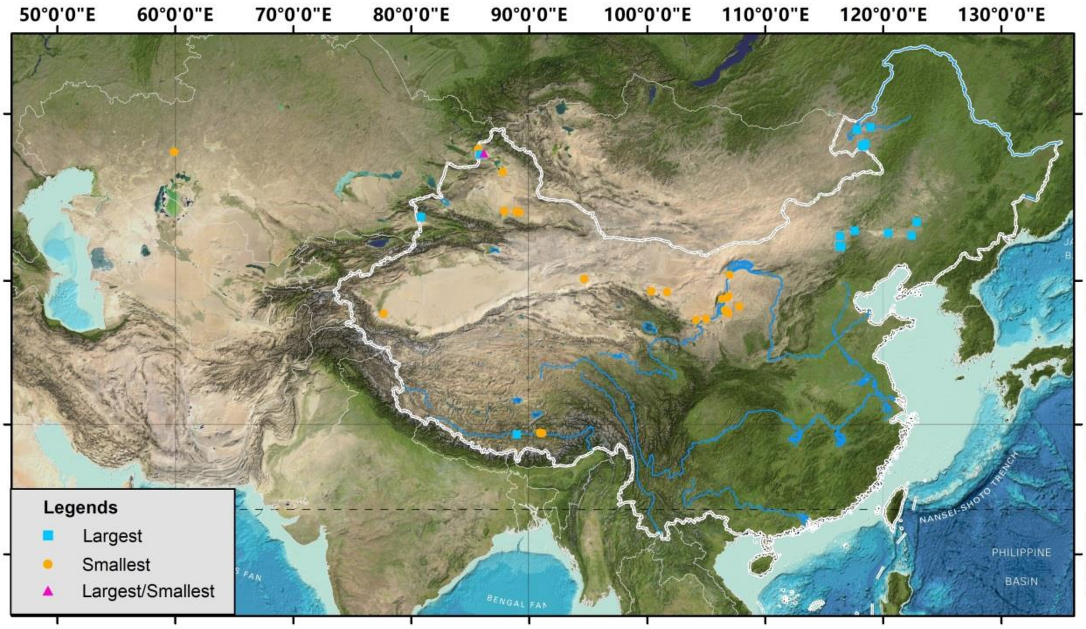

Figure S13 Seedling emergence days and plant height in common garden. (A) The days of seedling emergence of the large (C_L) and the small (C_S) group individuals. (B) The final plant height values of the C_L and the C_S group individuals. Statistical tests were performed and the *p* values are shown in each photo.

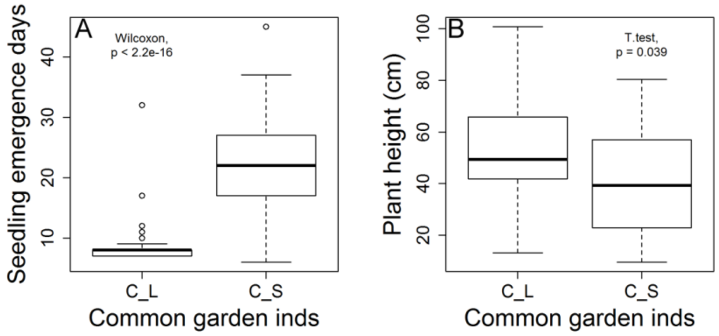

Figure S14 Important predictors selected by *leaps* R package for linear models of major axis (A) and TSW (B) at population level. The *leaps* function was used for the best subsets regression and all possible combination of the variables for major axis or TSW was tested according to the adjr2.

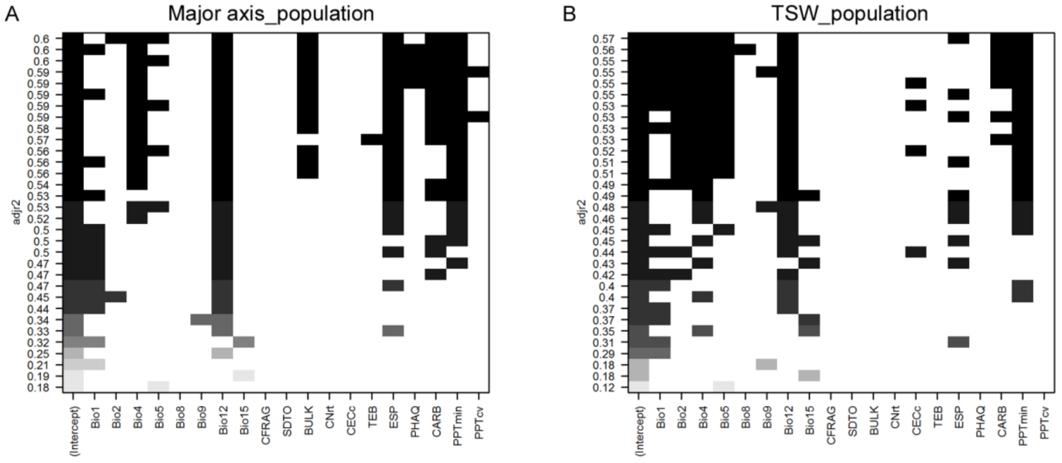

Supplement: erac231_suppl_Supplementary_Figures_S1-S4 [file erac231_suppl_supplementary_figures_s1-s4.pdf]
